# Endothelial SIRT3 deficiency predisposes brown adipose tissue to whitening in diet-induced obesity

**DOI:** 10.7150/ijbs.110741

**Published:** 2025-05-15

**Authors:** Qing Zhou, Zongshi Lu, Bowen Wang, Yuyan Wang, Li Li, Mei You, Lijuan Wang, Tingbing Cao, Dan Tong, Jie Xiang, Yu Zhao, Qiang Li, Aidi Mou, Wentao Shu, Hongbo He, Zhigang Zhao, Daoyan Liu, Zhiming Zhu, Peng Gao, Zhencheng Yan

**Affiliations:** Department of Hypertension and Endocrinology, Center for Hypertension and Metabolic Diseases, Daping Hospital, Army Medical University, and Chongqing Institute of Hypertension, Chongqing, China

**Keywords:** endothelium, BAT whitening, SIRT3, angiogenesis, angiocrine factor

## Abstract

Endothelial dysfunction and vascular rarefaction are supposed to be secondary to metabolic diseases, while recent evidence has revealed the primary roles of endothelium in initiating and accelerating metabolic disorders. Here, the effects and underlying mechanisms of endothelial SIRT3 in modulating the whitening of BAT during obesity progression were explored. Therefore, mice with global or BAT regional endothelium-specific Sirt3 knockout were constructed and fed with high-fat diet (HFD). The results showed that both global and BAT regional endothelium-specific Sirt3 knockout accelerated diet-induced weight gain, accompanied by glucose intolerance, insulin resistance, and BAT whitening. In vitro results revealed that the inhibition or knockdown of endothelial Sirt3 impeded palmitic acid-induced angiogenesis deficiency, while the overexpression of Sirt3 exhibited the opposite effects. Furtherly, endothelial Sirt3 overexpression ameliorated palmitic acid-induced adipocyte dysfunction and proinflammatory macrophages polarization in a paracrine way. Mechanistically, endothelial SIRT3 deficiency increased the acetylation of fatty acid synthase (FASN), which disturbed the fatty acid metabolism and thus, leading to angiogenesis insufficiency. Moreover, loss of SIRT3 promoted adipocytes dysfunction and proinflammatory macrophage polarization via CASP1-mediated pyroptosis. Endothelial SIRT3 loss contributed to diet-induced BAT whitening and obesity progression and thus, could be a therapeutic target in treating obesity and associated metabolic diseases.

## Introduction

Pathologic hypertrophy contributes to adipose tissue (AT) expansion, a process characterized by aberrant adipokine profiles, inflammatory responses, and vascular rarefaction^1^. Brown adipose tissue (BAT), the sole non-shivering thermogenetic tissue that embedded with dense vascular networks, plays crucial roles in modulating overall metabolism^2^. Therefore, the whitening of BAT, as indicated by adipocyte hypertrophy, lipid droplet accumulation, uncoupling protein 1 (UCP-1) suppression, and mitochondrial dysfunction, initiates and accelerates obesity progression^3^. While the contribution of brown adipocyte dysfunction to BAT whitening has been extensively explored, recent evidence has revealed the participation of nonadipocytes in regulating metabolic diseases^4^, ^5^. The traditional “one-way hypothesis” that endothelial dysfunction and vascular rarefaction are secondary pathological processes during obesity progression has been challenged by recent evidence indicating that endothelial changes precede obesogenic states and that manipulations of angiogenesis are sufficient to maintain metabolic homeostasis^4^-^6^.

Lining the inner layer of blood vessels, endothelial cells (ECs) are continuously exposed to multiple circulating factors and pathogenic stimuli that predispose them to damage and even senescence, which impairs angiogenesis and contributes to multiple pathological changes^7^, ^8^. ECs usually remain quiescent throughout adulthood, but upon ischemia or tissue injury, they switch to the proliferative state for tissue repair^9^. Therefore, the harmonious orchestration of EC subtypes, including migratory tip cells, proliferative stalk cells, as well as quiescent phalanx cells, guarantees angiogenesis^10^. Unlike in vascular smooth muscle cells, more than 85% of ATP in ECs is supplied by aerobic glycolysis instead of mitochondrial oxidative metabolism and this form of energy supply could further accelerate angiogenesis^11^. In addition to the high demand for energy, tremendous biomass synthesis is also needed during angiogenesis^11^. Although endothelial mitochondria represent only 2-5% of the cytoplasmic volume^12^, they work as biosynthesis hubs to provide intermediates for essential biomass synthesis in the process of cell proliferation and migration^13^, ^14^. Moreover, impaired mitochondrial function results in oxidative stress and thus leads to endothelial dysfunction and subsequent cardiovascular events^15^. Therefore, maintaining appropriate mitochondrial homeostasis is crucial for angiogenesis process.

In addition to being the passive conduits of blood transport, ECs also serve as a highly active endocrine niche that produces plenty of angiocrine factors, the crucial secretory factors that not only contribute to organ regeneration but also maintain the homeostasis of metabolism in other tissues^16^. In addition to boosting oxidative stress, impaired mitochondrial function is not only an inducer but also a consequence of senescence^7^. Endothelial senescence, a pathological process characterized by a senescence-associated secretory phenotype (SASP), leads to excessive cytokines production and results in subsequent angiogenesis inhibition^17^. Sirtuins, the NAD^+^-dependent deacetylases, have been proposed to be major regulators of cell senescence^7^. Sirtuin 3 (SIRT3), the major mitochondrial deacetylase, has been reported to be a key factor in modulating endothelial senescence^18^. SIRT3 contributes to the maintenance of mitochondrial metabolism and its deficiency results in mitochondrial dysfunction due to hyperacetylation of mitochondrial proteins^19^. Moreover, evidence has revealed that inactivation of SIRT3 causes DNA damage^20^, which therefore suggests the versatile effects of SIRT3 in modulating senescence. The significance of SIRT3 in modulating systemic metabolism, including insulin resistance and inflammation, has been extensively explored^21^, ^22^. Our previous study revealed that Sirt3 knockout exacerbates HFD-induced BAT whitening and obesity progression^23^. Nonetheless, another study showed that mice with adipocyte-specific Sirt3 knockout exhibit normal glucose and lipid metabolism, along with unchanged overall metabolism even after HFD feeding^24^, suggesting the participation of adipocyte-independent effects of SIRT3 during this process. Moreover, although recent evidence has revealed the significance of the endothelium in modulating obesity and its progression^4^, ^25^, the exact underlying mechanism still needs to be illustrated. Therefore, mice with global or BAT regional endothelium-specific Sirt3 knockout were constructed to investigate the effects of endothelial SIRT3 on BAT activity and obesity progression.

## Results

### Loss of endothelial Sirt3 exacerbated HFD-induced BAT whitening

**Figure 1:**
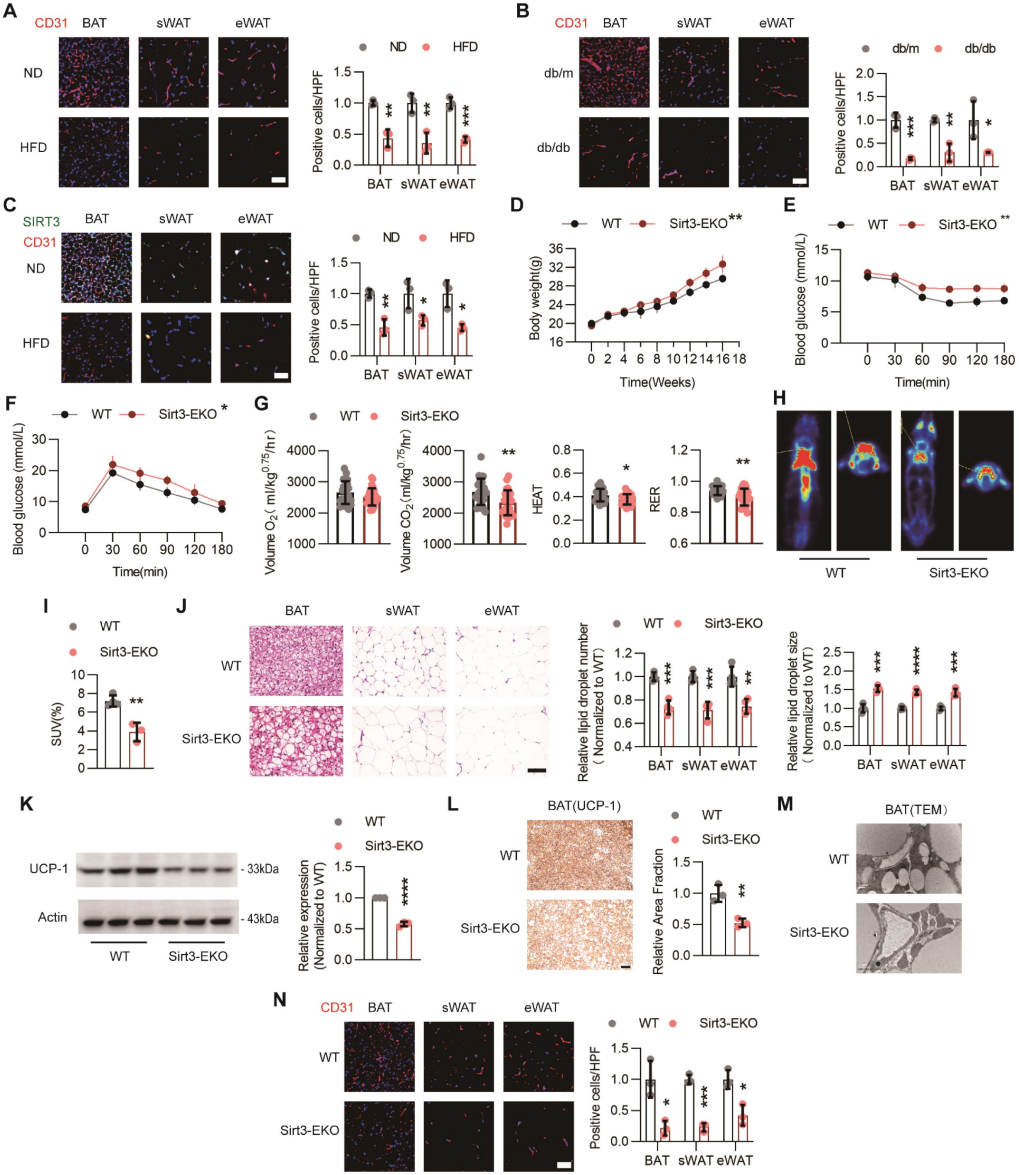
** Loss of endothelial SIRT3 exacerbated HFD-induced BAT whitening.** (**A**) Representative images of IF staining and corresponding quantitative results of CD31 (Red) in BAT, sWAT, and eWAT of C57 mice fed with ND or HFD (n=3). Scales bars, 50μm. (**B**) Representative images of IF staining and quantitative results of CD31 (red) in BAT, sWAT, and eWAT in db/m and db/db mice (n=3). Scales bars, 50μm. (**C**) Representative images of IF staining and quantitative results of CD31 (Red) and SIRT3 (Green) in BAT, sWAT, and eWAT of C57 mice fed with ND or HFD (n=3). Scales bars, 50μm. (**D**-**F**) The changes of body weight (**D**, n=4 or n=5**)**, ITT (**E**, n=3 or n=4**),** and IPGTT (**F**, n=4). (**G**) Volume O_2_, Volume CO_2_, Heat, and RER of Sirt3^flox/flox^-Tek-Cre mice (hereafter referred to as Sirt3-EKO mice) and Sirt3^flox/flox^ mice (hereafter referred to as WT mice) fed with HFD, detected by CLAMS (n=4). (**H**-**I**) Representative images of ^18^F-FDG uptake in BAT and quantitative results WT and Sirt3-EKO mice, detected by PET-CT scanning (n=3). (**J**) Representative images of H&E staining and quantitative results of the number and size of lipid droplets in BAT, sWAT, and eWAT of WT and Sirt3-EKO mice (n=4). Scales bars, 200μm. (**K**) Representative images of Western blots and quantitative results of UCP-1 in BAT of WT and Sirt3-EKO mice (n=3). β-actin served as a loading control. (**L**) Representative images of IHC staining and quantitative results of UCP-1 in BAT of WT and Sirt3-EKO mice (n=3). Scales bars, 200μm. (**M**) Representative images of TEM of BAT in WT and Sirt3-EKO mice (n=3). Scale bars, 1μm. (**N**) Representative images of IF staining and quantitative results of CD31 (Red) staining in BAT, sWAT and eWAT of WT an Sirt3-EKO mice (n=3). Scales bars, 50μm. ^*^P< 0.05, ^**^P< 0.01, ^***^P< 0.001, ^****^P< 0.0001 compared with ND (**A**,** C**), db/m (**B**), and WT (**D**-**L**, **N**). IF: immunofluorescence; BAT: brown adipose tissue; sWAT: subcutaneous white adipose tissue; eWAT: epididymal white adipose tissue; ND: normal diet; HFD: high fat diet; ITT: insulin tolerance test; IPGTT: Intraperitoneal glucose tolerance test; Volume O_2_: Oxygen consumption; Volume CO_2_: carbon dioxide production (Volume CO_2_); RER: respiratory exchange ratio; H&E: Hematoxylin and eosin; IIHC: immunohistochemical; TEM: Transmission Electron Microscope; UCP-1: uncoupling protein 1.

To evaluate the changes of vascular networks in adipose tissues during the obesity process, BAT, sWAT, and eWAT were isolated from normal diet (ND)- or HFD-treated C57 mice and further IF staining was performed. IF staining revealed that HFD treatment reduced the vascular density in both BAT and WAT, as indicated by decreased IF signals of CD31, an EC marker (Figure 1A). Similar rarefaction of vascular networks was also observed in db/db mice (Figure 1B). Accordingly, endothelial SIRT3 expression in BAT was also markedly reduced after HFD treatment (Figure 1C).

Next, the endothelium-specific Sirt3 knockout mice (Sirt3^flox/flox^-Tek-Cre mice, hereafter referred to as Sirt3-EKO mice) were constructed and endothelial Sirt3 deficiency were confirmed via Western blot (Figure S1A). As expected, long-term HFD treatment resulted in accelerated weight gain, especially in Sirt3-EKO mice (Figure 1D). Moreover, Sirt3-EKO mice exhibited worsen glucose intolerance and insulin resistance, when compared with their littermate controls (Sirt3^flox/flox^ mice, hereafter referred to as WT mice, Figure 1E-F). Furthermore, CLAMS was used to evaluate the changes of overall metabolism and we found that Sirt3-EKO mice exhibited a reduced overall metabolism, as suggested by decreased consumption of oxygen (Volume O_2_), generation of carbon dioxide (Volume CO_2_), HEAT, and respiratory exchange ratio (RER) (Figure 1G and Figure S1B). PET/CT scanning also revealed reduced uptake of ^18^F-FDG in BAT, suggesting the suppressed BAT activity in Sirt3-EKO mice (Figure 1H). Consistent with the overall metabolic changes, further H&E staining revealed obvious pathological hypertrophy of BAT and WAT in Sirt3-EKO mice (Fig 1I-J), along with reduced UCP-1 expression and increased mitochondria loss (Figure 1K-M). Consequently, endothelial Sirt3 knockout resulted in obvious vascular rarefaction in both BAT and WAT (Figure 1N). These results indicates that endothelial SIRT3 might play crucial roles in modulating diet-induced BAT whitening and obesity progression.

### Endothelial Sirt3 knockout in BAT accelerated HFD-induced BAT whitening

**Figure 2:**
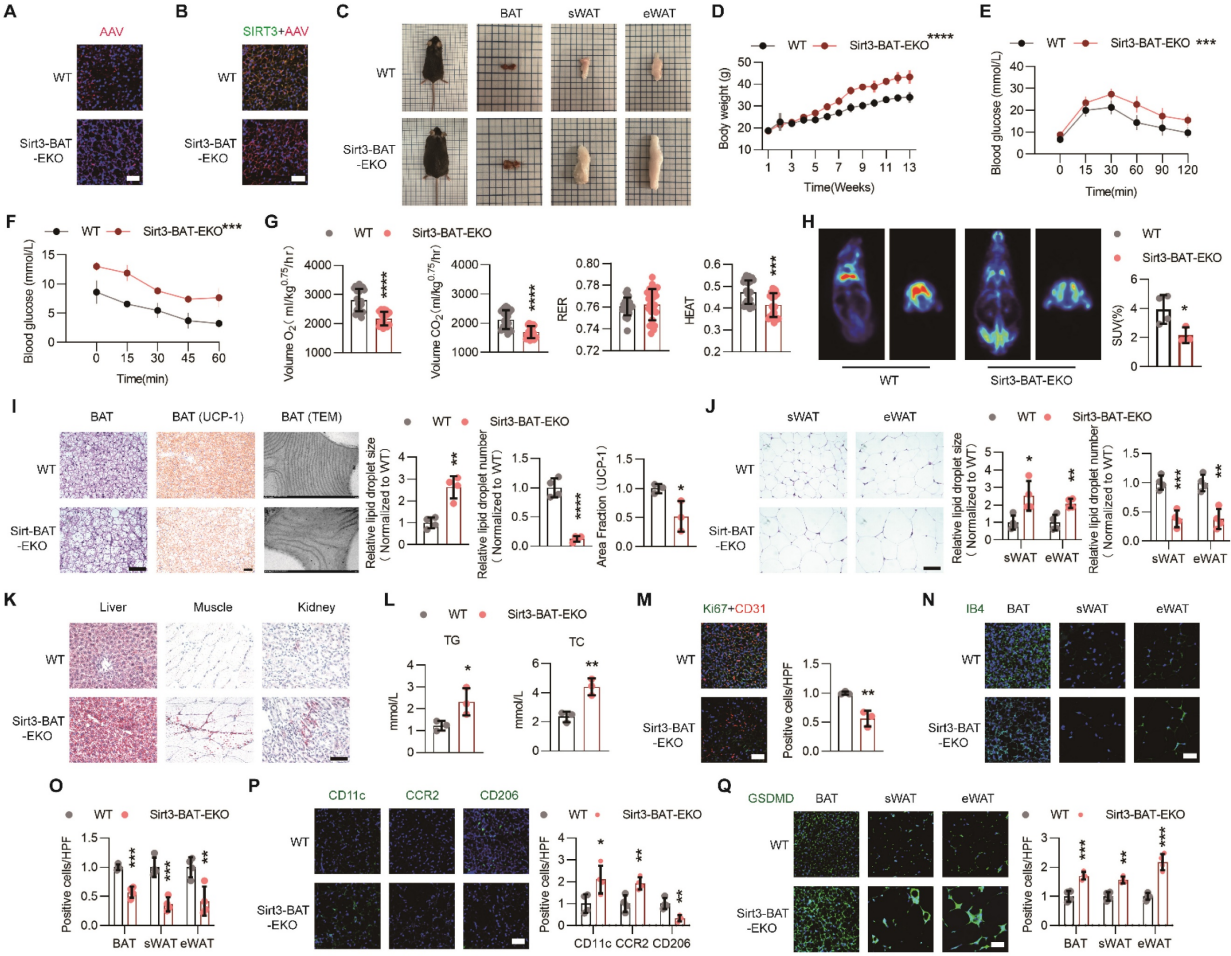
** Endothelial Sirt3 knockout in BAT accelerated HFD-induced BAT whitening.** (**A**) Representative images of IF staining of AAV (Red) in BAT from regional-specific endothelial Sirt3 knockout mice (hereafter referred to as Sirt3-BAT-EKO mice) and their control mice (hereafter referred to as WT mice) (n=4). Scales bars, 50μm. (**B**) Representative images of IF staining of AAV (Red) and SIRT3 (Green) in BAT of WT and Sirt3-BAT-EKO mice (n=4). Scales bars, 50μm. (**C**) Representative images of the whole appearance, BAT, sWAT, and eWAT of WT and Sirt3-BAT-EKO mice at the end of the HFD intervention. (**D-F**) The changes of body weight (**D**, n=6 or n=8), blood glucose levels during the IPGTT (**E**, n=6 or n=8**)** and ITT (**F**, n=3 or n=4) of WT and Sirt3-BAT-EKO mice fed with HFD. (**G**) the changes of Volume O_2_, Volume CO_2_, RER, and Heat of WT and Sirt3-BAT-EKO mice fed with HFD, detected by CLAMS (n=6). (**H**) Representative images of ^18^F-FDG uptake in BAT in WT and Sirt3-BAT-EKO mice, detected by PET-CT scanning (n=3 or n=4). (**I**) Representative images of H&E staining, IHC staining of UCP-1, TEM of mitochondria, and quantitative results of lipid droplets and IHC staining, of BAT in WT and Sirt3-BAT-EKO mice (n=3 or n=4). Scales bars, 200μm or 200nm (TEM). (**J**) Representative images of H&E staining and quantitative results of the number or size of lipid droplets in BAT from WT and Sirt3-BAT-EKO mice (n=4). Scales bars, 200μm. (**K**) Representative images of Oil Red O staining of the liver, muscle, and kidney from WT and Sirt3-BAT-EKO mice (n=3). Scales bars, 200μm. (**L**) The levels of serum Triglyceride (TG) and Total Cholesterol (TC) in WT and Sirt3-BAT-EKO mice (n=3). (**M**) Representative images of IF staining (left) and quantitative results (right) of Ki67 (Green) and CD31 (Red) of BAT in WT and Sirt3-BAT-EKO mice (n=3). Scales bars, 50μm. (**N-Q**) Representative images of IF staining and quantitative results of IB4 (green, **N**, **O**), CD11c (green, **P**), CCR2 (green, **P**), CD206 (green, **P**), and GSDMD (green, **Q**) of BAT in WT and Sirt3-BAT-EKO mice (n=3). Scales bars, 50μm. ^*^P< 0.05, ^**^P< 0.01, ^***^P< 0.001, ^****^P< 0.0001 compared with WT mice (**D**, **E**, **F**, **G**,** H**, **I**,** J**, **L**,** M**, **O**,** P**, **Q**). AAV: Adeno-associated Virus; TG: Triglyceride; TC: Total Cholesterol; ITT: insulin tolerance test; IPGTT: Intraperitoneal glucose tolerance test; IB4: Isolectin B4; IF: immunofluorescence; BAT: brown adipose tissue; sWAT: subcutaneous white adipose tissue; eWAT: epididymal white adipose tissue; ND: normal diet; HFD: high fat diet; Volume O_2_: Oxygen consumption; Volume CO_2_: carbon dioxide production (Volume CO_2_); RER: respiratory exchange ratio; H&E: Hematoxylin and eosin; IIHC: immunohistochemical; TEM: Transmission Electron Microscope; UCP-1: uncoupling protein 1.

To evaluate the effects of local endothelial SIRT3 on BAT thermogenesis and overall metabolism, mice with BAT regional endothelium-specific SIRT3 knockout were constructed using recombinant AAV-mediated gene delivery (hereafter referred to as Sirt3-BAT-EKO mice) 26. Successful delivery was verified in frozen sections of BAT (Figure 2A). Furtherly, IF staining revealed the efficiency of AAV-mediated endothelial Sirt3 knockout, as demonstrated by the absence of colocalization of AAV and SIRT3 fluorescence, which were consistent with the results of Western blot (Figure 2B and Figure S2A).

As expected, long-term HFD feeding accelerated weight gain in Sirt3-BAT-EKO mice without affecting food intake (Figure 2C-D and Figure S2B). Moreover, the glucose tolerance and insulin sensitivity significantly declined in Sirt3-BAT-EKO mice (Figure 2E-F). CLAMS results also revealed the reduced Volume O_2_, Volume CO_2_, and HEAT in Sirt3-BAT-EKO mice (Figure 2G and Figure S2C), indicating that endothelial SIRT3 in BAT affected the overall metabolism. Surprisingly, the uptake of ^18^F-FDG was obviously decreased in BAT but increased in skeletal muscle, especially in Sirt3-BAT-EKO mice (Figure 2H and Figure S2D), suggesting that skeletal muscle might replace BAT to absorb glucose via an unknown mechanism after SIRT3 disappeared in BAT vasculature. At the end of HFD treatment, Sirt3-BAT-EKO mice displayed obvious BAT whitening (Figure 2I), accompanied with abnormal expansion of sWAT and eWAT (Figure 2J). Consistently, ectopic lipid depositions in liver, kidney, as well as skeletal muscle, were more obvious in Sirt3-BAT-EKO mice, with an increased serum Triglyceride (TG) and total cholesterol (TC) (Figure 2K-L), suggesting that the loss of Sirt3 in BAT vascular endothelium resulted in abnormal lipid metabolism. Moreover, Sirt3 knockout resulted in obvious endothelial senescence, as indicated by reduced colocalization of Ki67 and CD31 signals (Figure 2M). Consistently, vascular rarefaction was more severe in both BAT and WAT in Sirt3-BAT-EKO mice (Figure 2N-O), accompanied with excessive infiltration of proinflammatory macrophages and increased pyroptosis in BAT, as indicated by reduced signal of CD206 and increased signals of CD11c, CCR2, and Gasdermin D (GSDMD) (Figure 2P-Q). These results indicate that even local SIRT3 deficiency limited to endothelial cells in BAT is sufficient to induce systemic metabolic abnormalities, further emphasizing the importance of the BAT vascular network in maintaining metabolic homeostasis.

### Endothelial SIRT3 deficiency impeded angiogenesis

**Figure 3:**
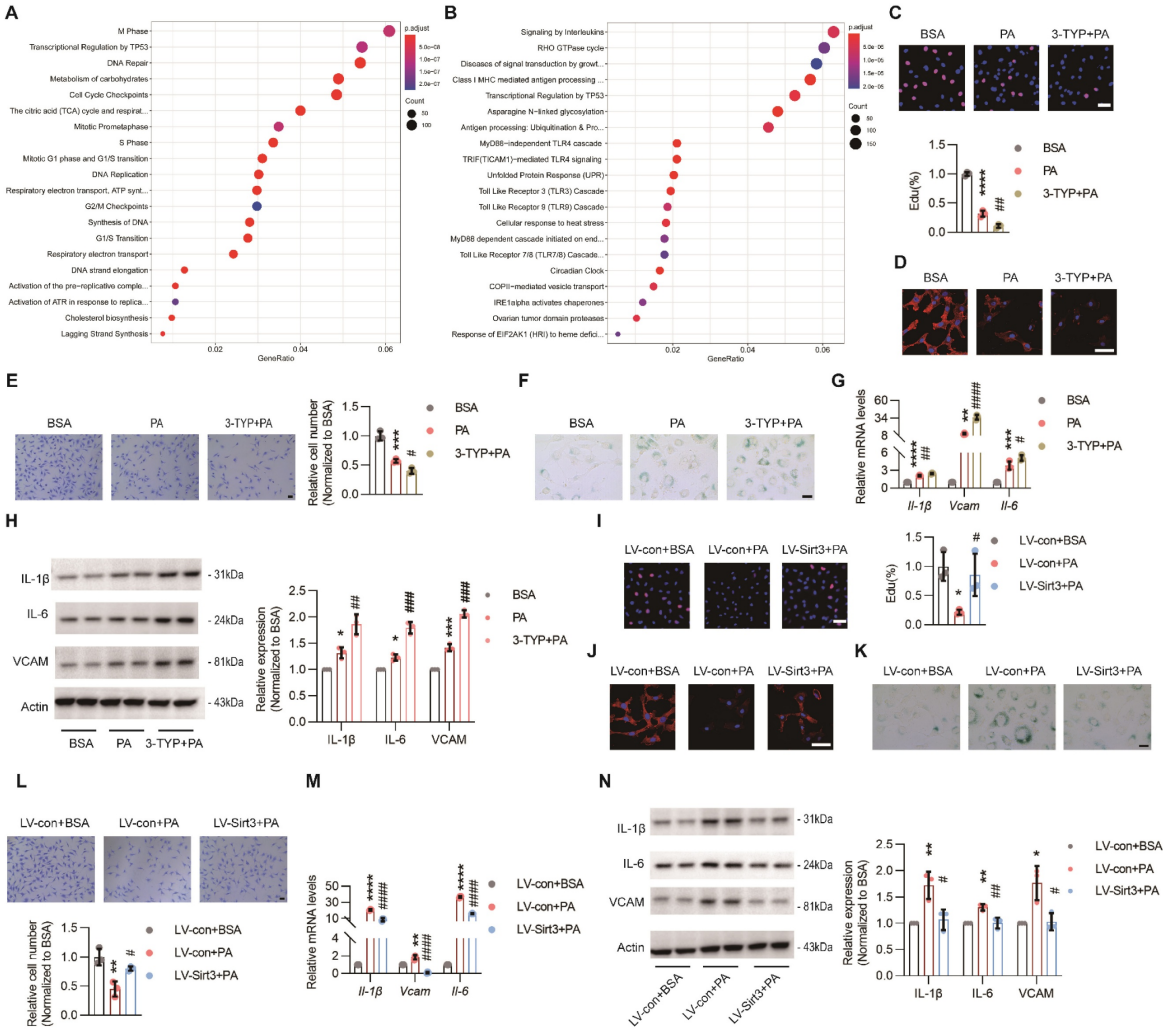
** Endothelial SIRT3 deficiency impeded angiogenesis.** (**A-B**) Representative images of Reactome analysis of down-regulated (**A**) and up-regulated (**B**) pathways in HUVECs (hereafter referred to as ECs) treated with BSA (150μM) or PA (150μM), detected by RNA sequencing (n=3). (**C**-**F**) Representative images and quantitative results of EdU staining (**C**), F-actin (**D**), transwell (**E**), and SA-β-galactosidase (**F**) staining in ECs treated with BSA, PA, or 3-TYP (50μM) +PA (n=3). Scale bars, 50μm. (**G**) The mRNA levels of *Il-1β*, *Vcam*, and *Il-6* in ECs treated with BSA, PA, or 3-TYP+PA (n=3). β-actin served as a loading control. (**H**) Representative images of Western blots and quantitative results of the indicated cytokines in ECs treated with BSA, PA, or 3-TYP+PA (n=3). β-actin served as a loading control. (**I**-**L**) Representative images and quantitative results of EdU staining (**I**), F-actin (**J**), SA-β-galactosidase (**K**), and transwell (**F**) assays in ECs treated with LV-con+BSA, LV-con+PA, or LV-Sirt3+PA (n=3). Scales bars, 50μm. (**M**) The mRNA levels of *Il-1β*, *Vcam*, and *Il-6* in ECs treated with LV-con+BSA, LV-con+PA, or LV-Sirt3+PA (n=3). β-actin served as a loading control. (**N**) Representative images of Western blots and quantitative results of indicated cytokines in ECs treated with LV-con+BSA, LV-con+PA, or LV-Sirt3+PA (n=3). β-actin served as a loading control. ^*^P< 0.05, ^**^P< 0.01, ^***^P< 0.001, ^****^P< 0.0001 compared with BSA (**C**, **E**, **G**, **H**), and LV-con+BSA (**I**, **L**, **M**, **N**).^ #^*P*< 0.05, ^##^*P*< 0.01, ^###^*P*< 0.001, ^####^*P*< 0.0001 compared with PA (**C**, **E**, **G**, **H**) and LV-con+PA (**I**, **L**, **M**, **N**). HUVECs: Human umbilical vein endothelial cells; BSA: bovine serum albumin; PA: palmitic acid; IL-6: Interleukin 6; IL-1β: Interleukin 1β; VCAM: Vascular cell adhesion molecule.

To explore the underlying mechanism of pro-obesogenic effects of endothelial SIRT3, HUVECs (hereafter referred to as ECs) were used. First, RNA sequencing (RNA-seq) data revealed that PA treatment could result in obvious EC senescence, as indicated by impaired DNA repair, reduced DNA synthesis, and downregulated genes concerning cell phases, including S phase, M phase, G1 phase, and G1/S transition (Figure 3A and Figure S3A), as well as upregulated cell stress and proinflammatory pathways, such as unfolded protein response (UPR), signaling by interleukins, MyD88-independent TLR4 cascade, and TICAM1-mediated TLR4 cascade (Figure 3B and Figure S3B). Consistent with these results, PA treatment dampened angiogenesis process, as suggested by decreased signals of EdU and F-actin staining, reduced migratory cells and tube formation, and down-regulated pro-angiogenic factors, including Vascular endothelial growth factor receptor 2 (VEGFR2), NOTCH1, and platelet-derived growth factor receptor β (PDFGRβ). Moreover, all these detrimental effects were further exacerbated by 3-TYP, a selective inhibitor of SIRT3 (Figure 3C-E and Figure S3C-D). Additionally, SA-β-galactosidase and SASP cytokines, including IL-6, Vcam, IL-1β, and TNF-α, were remarkably up-regulated due to PA treatment and were further exacerbated by 3-TYP (Figure 3F-H). On the contrary, all these detrimental and pro-senescent effects of PA were significantly improved and even reversed after lentivirus-mediated Sirt3 overexpression (Figure 3I-N and Figure S3F-G), suggesting that endothelial SIRT3 was a chief guard that protected against the loss of angiogenesis ability in parallel with cell senescence.

### Loss of endothelial SIRT3 exacerbated mitochondrial dysfunction and disturbance of fatty acid metabolism

**Figure 4:**
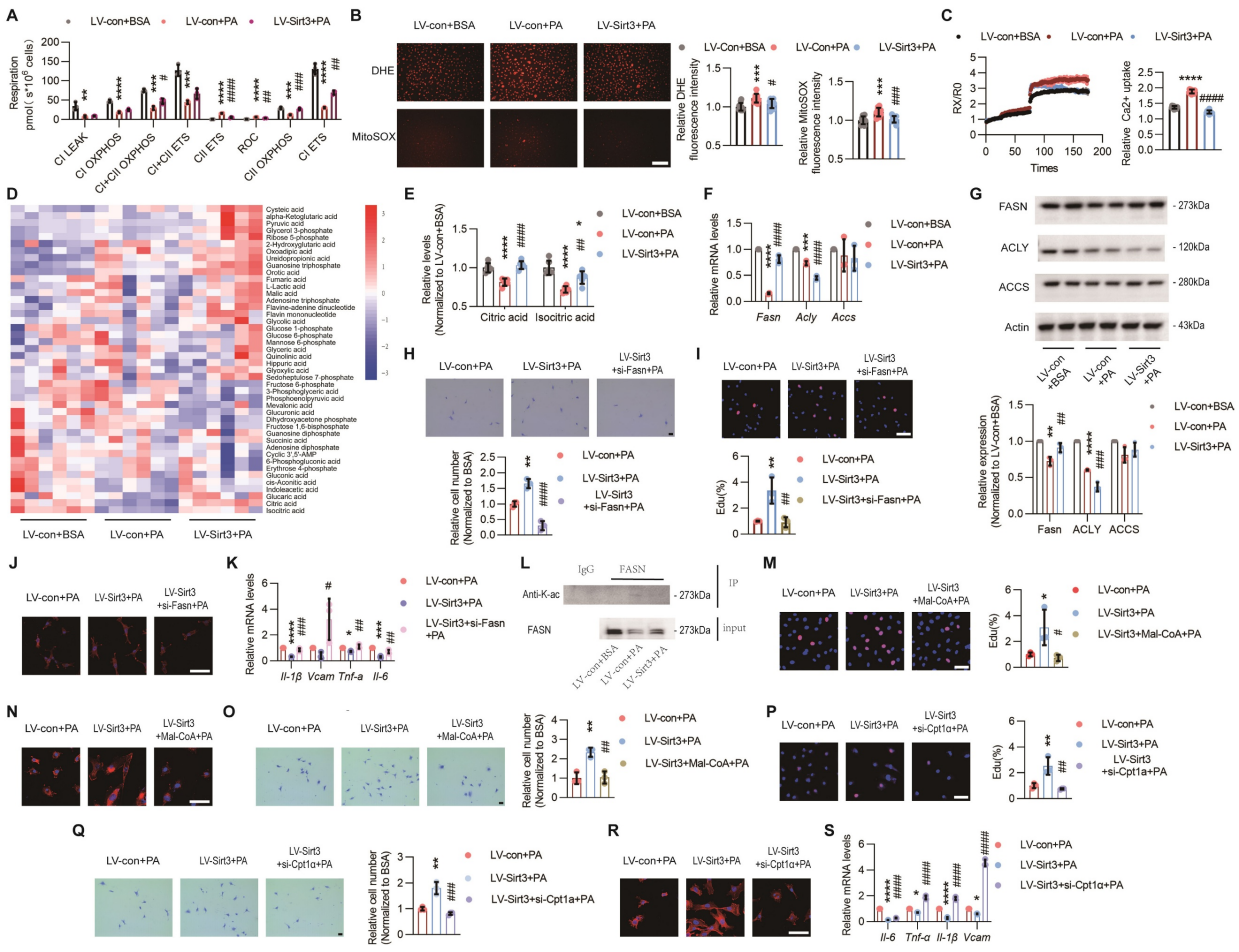
** Loss of endothelial SIRT3 exacerbated mitochondrial dysfunction and disturbance of fatty acid metabolism.** (**A-C**) The changes of mitochondrial respiration indices, including CⅠ leak, CⅠ OXPHOS, CⅠ+Ⅱ OXPHOS, CⅠ+Ⅱ ETS, CⅡ ETS, and ROC, detected by O2k (**A**, n=3), cytosolic and mitochondrial ROS measured by DHE and MitoSOX fluorescent staining and individual quantitative results (**B**, n=3), and changes of mitochondrial Ca^2+^ that labeled with Rhod-2 AM (**C**, n=3) in ECs treated with LV-con+BSA, LV-con+PA, or LV-Sirt3+PA. Scales bars, 200μm. (**D-E**) The changes of mitochondrial metabolites profile detected via targeted metabolomics (**D**, n=6), especially Citric acid and Isocitric acid (**E**), in ECs treated with LV-con+BSA, LV-con+PA, or LV-Sirt3+PA (n=6). (**F**) The mRNA levels of *Fasn*, *Acly*, and *Accs* in ECs treated with LV-con+BSA, LV-con+PA, or LV-Sirt3+PA (n=3). β-actin served as a loading control. (**G**) Representative images of Western blots and quantitative results of indicated cytokines in ECs treated with LV-con+BSA, LV-con+PA, or LV-Sirt3+PA (n=3). β-actin served as a loading control. (**H**-**J**) Representative images and the corresponding quantitative results of transwell (**H**), EdU staining (**I**), F-actin (**J**), and mRNA levels of the indicated cytokines (**K**) in ECs treated with LV-con+BSA, LV-con+PA, or LV-Sirt3+PA (n=3). Scales bars, 50μm. (**L**) Representative blots of FASN and immunoblotted by Acetyllysine in RAW264.7 cells treated with LV-con+BSA, LV-con+PA, or LV-Sirt3+PA (n=3). (**M**-**O**) Representative images and the corresponding quantitative results of EdU staining (**M**), F-actin (**N**), and transwell (**O**) assays of ECs treated with LV-con+PA, LV-Sirt3+PA, or LV-Sirt3+Mal-CoA (20 μM)+PA (n=3). Scales bars, 50μm. (**P**-**S**) Representative images and quantitative results of EdU staining (**P**), transwell assays (**Q**), F-actin (**R**), and mRNA levels of the indicated cytokines (**S**) in ECs treated with LV-con+PA, LV-Sirt3+PA, or LV-Sirt3+si-Cpt-1a+PA (n=3). Scales bars, 50μm. ^*^P< 0.05, ^**^P< 0.01, ^***^P< 0.001, ^****^P< 0.0001 compared with LV-con+BSA (**A**, **B**, **C**, **E**, **F**, **G**), and LV-con+PA (**H**, **I**,** K**, **M**, **O**, **P**, **Q**, **S**).^ #^*P*< 0.05, ^##^*P*< 0.01, ^###^*P*< 0.001, ^####^*P*< 0.0001 compared with LV-con+PA (**A**, **B**, **C**, **E**, **F**, **G**), and LV-con+Sirt3 (**H**, **I**,** K**, **M**, **O**, **P**, **Q**, **S**). BSA: bovine serum albumin; PA: palmitic acid; CⅠ leak: Complex Ⅰ leak; CⅠ OXPHOS: Complex Ⅰ oxidative phosphorylation; CⅠ+Ⅱ OXPHOS: Complex Ⅰ and Complex Ⅱ oxidative phosphorylation; CⅠ+Ⅱ ETS: Complex Ⅰ and Complex Ⅱ electron transport system; CⅡ ETS: Complex Ⅱ electron transport system; ROC: Residual oxygen consumption; O2k: Oxygraph-2k; FASN: fatty acid synthase; ACLY: ATP citrate lyase; ACCS: acetyl-CoA Carboxylase; IL-6: Interleukin 6;; IL-1β: Interleukin 1β; VCAM: Vascular cell adhesion molecule.

Rather than being the powerhouse, endothelial mitochondria are indeed the biosynthesis and signaling hubs during angiogenesis^13^. While the RNA sequencing results showed that the TCA cycle and electron transport chain (ETC) pathways were strongly downregulated after PA treatment (Figure 2A and Figure S2A), further in vitro experiments also verified that PA-induced mitochondrial dysfunction, as evidenced by impaired mitochondrial respiration, increased ROS overproduction, and excessive mitochondrial calcium uptake, were obviously reversed after Sirt3 overexpression (Figure 4A-C), indicating the crucial roles of SIRT3 in maintaining endothelial mitochondrial homeostasis.

The endothelial performance has been reported to be regulated by the intermediates of mitochondrial TCA cycle^22^. Therefore, we performed targeted metabolomics and the results showed that the levels of citric acid (Citrate) and isocitric acid (Isocitrate) were significantly reduced after PA treatment, whereas only Citrate was completely restored after Sirt3 overexpression (Figure 4D-E and Figure S4A). Citrate acts as the crucial precursor of fatty acid synthesis^27^, a process that is crucial for angiogenesis^28^. Hence, the expressions of the rate-limiting enzymes of fatty acid synthesis, including ATP citrate lyase (ACLY), acetyl-CoA Carboxylase (ACCS), and fatty acid synthase (FASN), were determined. The results showed that only FASN could be restored by Sirt3 overexpression upon PA treatment (Figure 4F-G).

To determine whether FASN was a critical downstream target of SIRT3, we constructed a plasmid containing Fasn siRNA and found that the proangiogenic effects of Sirt3 were dampened after Fasn interference, as evidenced by impaired angiogenesis process and up-regulated SASP factors (Figure 4H-K and Figure S4C-D). Next, the acetylation levels of FASN were evaluated through co-IP and we found that the acetylated FASN increased after PA pretreatment while decreased due to Sirt3 overexpression (Figure 4K). Increased acetylation destabilizes FASN by ubiquitin‒proteasome-mediated degradation, thus leading to the accumulation of malonyl-CoA (Mal-CoA)^28^, the robust endogenous inhibitor of Carnitine Palmitoyltransferase 1a (CPT-1a)^14^. Here, in addition to siRNA-mediated Cpt-1a knockdown, inhibiting CPT-1a via Mal-CoA or etomoxir sodium salt (Eto), also obviously weakened the proangiogenic effects of Sirt3 overexpression (Figure 4L-S and Figure S4E-O), indicating that the proangiogenic effects of SIRT3 might be mediated by mitochondrial fatty acid metabolism.

### Loss of endothelial SIRT3 exacerbated adipocyte dysfunction and proinflammatory macrophages polarization in a paracrine way

**Figure 5:**
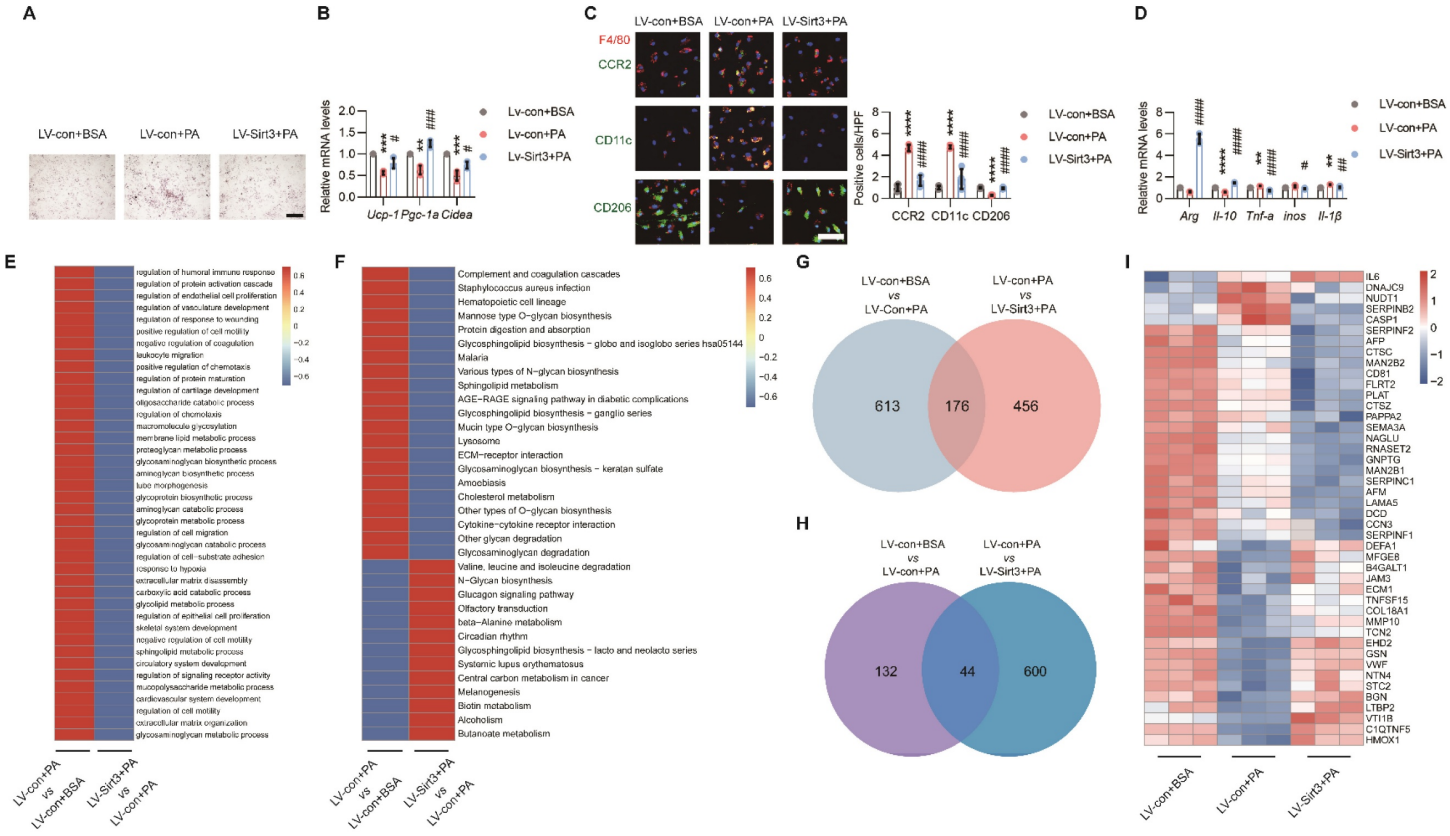
** Loss of endothelial SIRT3 exacerbated adipocyte dysfunction and pro-inflammatory macrophages polarization in a paracrine way. (A-B)** Representative images of lipid droplets formation **(A)** and mRNA levels of *Ucp-1*, *Pgc-1α*, and *Cidea* **(B)** in terminally differential brown 3T3-L1 adupocytes treated with the CM of LV-con+BSA, LV-con+PA, or LV-Sirt3+PA-pretreated ECs (n=3). Scales bars, 200μm. β-actin served as a loading control. **(C)** Representative images of IF staining (left) and quantitative results (right) of CD11c (Green), CD206 (Green), CCR2 (Green), and F4/80 (Red) in BMDMs treated with the CM of LV-con+BSA, LV-con+PA, or LV-Sirt3+PA-pretreated ECs (n=3). Scales bars, 50μm. **(D)** The mRNA levels of *Arg*, *Il-10*, *Tnf-α*, *inos*, and *Il-1β* in BMDMs treated with the CM of LV-con+BSA, LV-con+PA, or LV-Sirt3+PA-pretreated ECs (n=3). β-actin served as a loading control. **(E-F)** Representative images of cluster analysis of BP **(E)** and KEGG analysis **(F)** in CM of ECs treated with LV-con+BSA, LV-con+PA, or LV-Sirt3+PA, detected via proteomics (n=3). The data were normalized to LV-con+BSA. **(G)** Representative image of Venn diagram of CM from ECs treated with LV-con+BSA, LV-con+PA, or LV-Sirt3+PA, detected via proteomics (n=3). **(H-I)** Representative images of the Venn diagram **(H)** and Heat map **(I)** of secreted proteins (Angiocrine factors) of CM from ECs treated with LV-con+BSA, LV-con+PA, or LV-Sirt3+PA, detected by proteomics (n=3). ^**^P< 0.01, ^***^P< 0.001, ^****^P< 0.0001 compared with the LV-con+BSA (B, C, D).^ ##^*P*< 0.01, ^###^*P*< 0.001, ^####^*P*< 0.0001 compared with the LV-con+PA **(B, C, D)**. BSA: bovine serum albumin; PA: palmitic acid; UCP-1: uncoupling protein 1; PGC-1α: Peroxisome proliferator-activated receptor-gamma coactivator 1 α; Cidea: Cell death inducing DFFA like effector A; CM: conditional medium; BMDMs: bone marrow derived macrophages; Arg: Arginase; IL-10: Interleukin 10; iNOS: inducible Nitric oxide synthase.

As ECs also deploy multiple angiocrine factors that participate in organ regeneration and metabolism maintenance^16^. To explore the possible effects of endothelial SIRT3 on surrounding cells, the terminally differentiated brown 3T3-L1 adipocytes and BMDMs were used and treated with the conditional medium (CM) of ECs. As expected, the CM of PA-pretreated ECs promoted lipid accumulation and suppressed thermogenic genes in brown 3T3-L1 adipocytes (Figure 5A-B). For BMDMs, the CM of PA-pretreated ECs promoted the polarization of proinflammatory M1 macrophages, as indicated by increased signals of CD11c^+^F4/80^+^ and CCR2^+^F4/80^+^ and overproduction of proinflammatory cytokines (Figure 5C-D). However, all these effects were improved after endothelial Sirt3 overexpression (Figure 5A-D). In addition to PA, the CM of LPS-, Ang II-, or ox-LDL-pretreated ECs exhibited similar effects on brown 3T3-L1 adipocytes and BMDMs (Figure S5A-L), which, therefore, indicating the crucial roles of endothelial angiocrine function in modulating adipocytes and macrophages.

Next, to explore the underlying mechanism, the CMs of ECs that treated with lentivirus and the controls were collected for further proteomic analysis. Cluster analysis revealed upregulated complement and coagulation cascades, as well as cytokine‒cytokine receptor interactions, in ECs treated with PA (Figure 5E-F). Further secretome and Venn analysis revealed that a total of 176 proteins exhibited changes among these groups (Figure 5G and Figure S6A), of which only 44 secretory proteins exhibited opposite changes (Figure 5H-I and Fig S6B).

### Endothelial SIRT3 deficiency induced pyroptosis in surrounding cells

**Figure 6:**
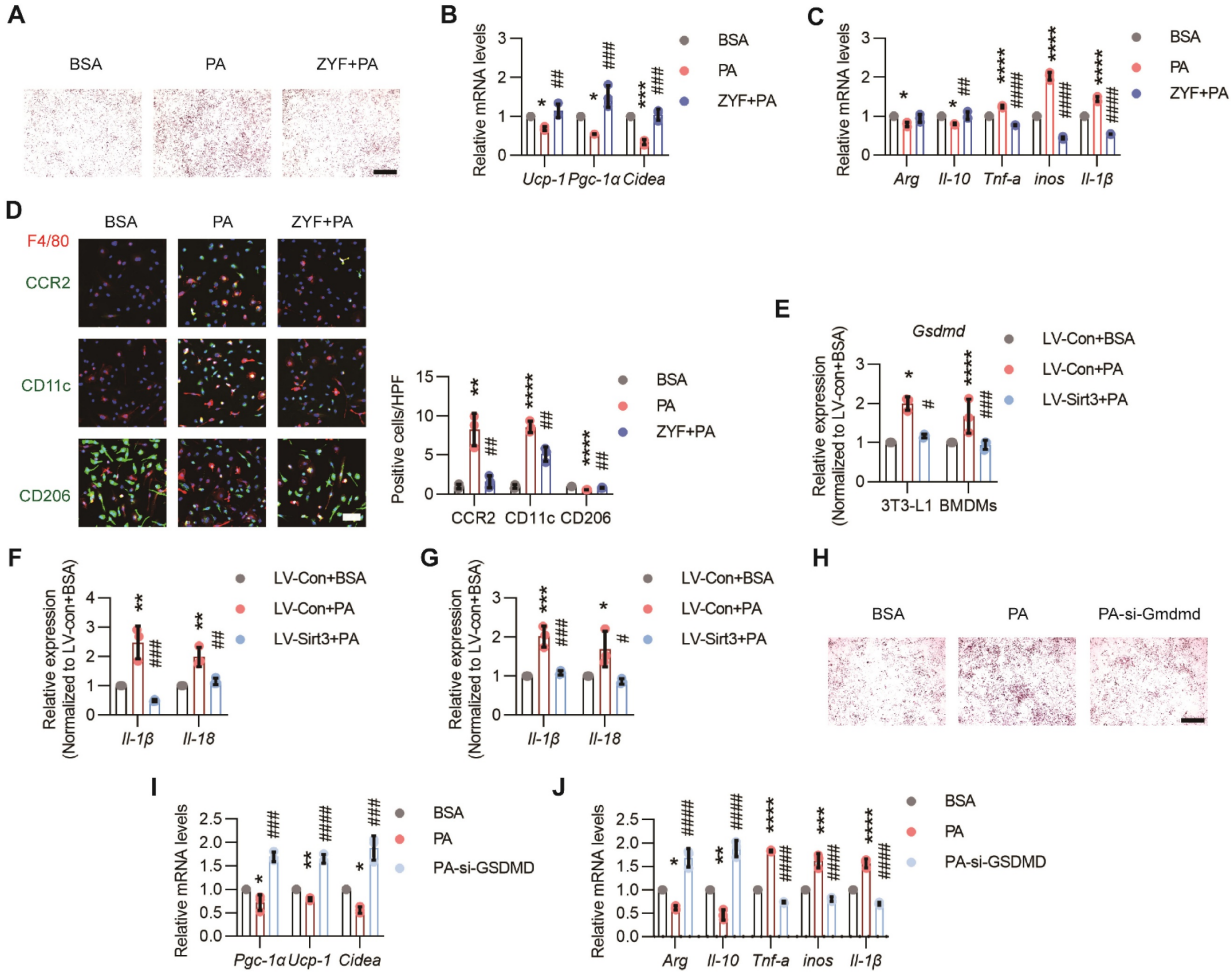
** Endothelial SIRT3 deficiency induced pyroptosis in surrounding cells.** (**A-B**) Representative images of lipid droplets formation and the mRNA levels of *Ucp-1*, *Pgc-1α*, and *Cidea* in terminally differential brown 3T3-L1 adipocytes treated with the CM of BSA, PA, or ZYF (10μM) + PA-retreated ECs (n=3). Scales bars, 200μm. β-actin served as a loading control. (**C**) The mRNA levels of *Arg*, *Il-10*, *Tnf-α*, *inos*, and *Il-1β* in BMDMs treated with the CM of BSA-, PA-, or ZYF+PA-pretreated ECs (n=3). β-actin served as a loading control. (**D**) Representative images of IF staining (left) and quantitative results (right) of CD11c (Green), CD206 (Green), CCR2 (Green), and F4/80 (Red) in BMDMs treated with the CM of BSA-, PA-, or ZYF+PA-pretreated ECs (n=3). Scales bars, 50μm. (**E**) The mRNA levels of *Gsdmd* in terminally differential brown 3T3-L1 adipocytes or BMDMs treated with the CM of LV-con+BSA, LV-con+PA, or LV-Sirt3+PA-pretreated ECs (n=3). β-actin served as a loading control. (**F**-**G**) The mRNA levels of *Il-1*β and *Il-18* in BMDMs (**F**) and terminally differential 3T3-L1 adipocytes (**G**) treated with the CM of LV-con+BSA, LV-con+PA, or LV-Sirt3+PA-pretreated ECs (n=3). β-actin served as a loading control. (**H**-**I**) Representative images of lipid droplets formation **(H)** and the mRNA levels of *Ucp-1*, *Pgc-1α*, and* Cidea* **(I)** in si-con or si-Gsdmd-pretreated terminal differential brown 3T3-L1 adipocytes that treated with the CM of PA-pretreated ECs (n=3). Scales bars, 200μm. β-actin served as a loading control. (**J**) The mRNA levels of *Arg*, *Il-10*, *Tnf-α*, *inos*, and *Il-1β* in si-con- or si-Gsdmd-pretreated RAW264.7 cells treated with the CM of PA-pretreated ECs (n=3). β-actin served as a loading control. ^*^P< 0.05, ^**^P< 0.01, ^***^P< 0.001, ^****^P< 0.0001 compared with BSA (**B**, **C**, **D**,** I**,** J**), LV-con+BSA (**I**, **J**, **K**, **M**, **N**).^ #^*P*< 0.05,^ ##^*P*< 0.01, ^###^*P*< 0.001, ^####^*P*< 0.0001 compared with PA (**B**, **C**, **D, I**,** J**), LV-con+PA (**E**, **F**, **G**, **I**, **J**). ZYF: Z-YVAD-FMK; GSDMD: Gasdermin D; IL-18: Interleukin 18; BSA: bovine serum albumin; PA: palmitic acid; UCP-1: uncoupling protein 1; PGC-1α: Peroxisome proliferator-activated receptor-gamma coactivator 1 α; Cidea: Cell death inducing DFFA like effector A; IL-1β: Interleukin 1β; IL-18: Interleukin 18.

Among the secretory proteins produced by ECs, Caspase 1 (CASP1), one of the main culprits of adipocyte dysfunction and inflammation^29^, ^30^, was simultaneously elevated after PA treatment while decreased by Sirt3 overexpression (Figure 5I and Figure S6B). Indeed, Z-YVAD-FMK (ZYF), an irreversible caspase-1 inhibitor, could obviously alleviate lipid droplets accumulation and restore thermogenetic genes expression in brown 3T3-L1 adipocytes (Figure 6A-B). Moreover, the proinflammatory effects of PA pretreated ECs-derived CM were also significantly improved by ZYF (Figure 6C-D).

Pyroptosis, the newly defined cell death process regulated by CASP1, has been proposed to be a crucial process in various diseases, including inflammation and metabolic diseases^31^. Here we found that the mRNA levels of Gsdmd in brown 3T3-L1 adipocytes and BMDMs were obviously elevated when these cells were treated with the CM of PA-pretreated ECs, whereas reduced after endothelial Sirt3 overexpression (Figure 6E). Moreover, the pyroptosis factors, such as Il-1β and Il-18, as well as cell death as indicated by CCK8 assessment, in brown 3T3-L1 adipocytes and BMDMs were also apparently increased when treated with PA pre-treated ECs and decreased after endothelial Sirt3 overexpression, respectively (Figure 6F-G and Figure S7A-B). Additionally, the pro-whitening and pro-pyroptosis effects of PA-treated ECs were obviously alleviated after Gsdmd interference, as suggested by the decreased accumulation of lipid droplets, increased expression of thermogenesis genes, and enhanced cell viability (Figure 6H-I and Figure S7D). Accordingly, similar results were obtained for Gsdmd-interfered RAW264.7 cells, as indicated by the reduced mRNA levels of proinflammatory cytokines and increased cell viability (Figure 6J and Figure S7F), suggesting the participation of pyroptosis in mediating the proinflammatory effects of dysfunctional ECs.

## Discussion

**Figure 7:**
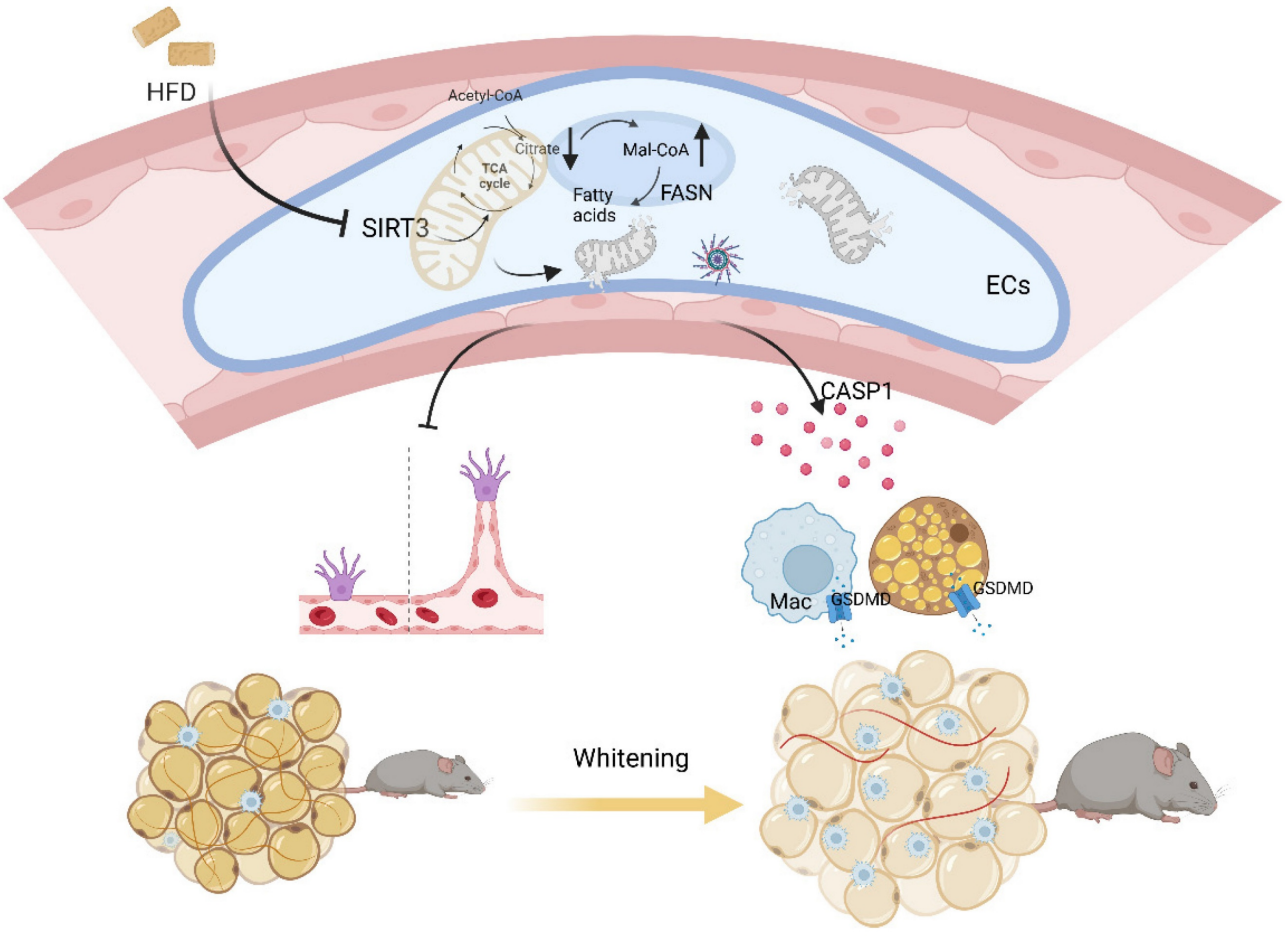
** Working model.** Loss of endothelial SIRT3 resulted in vascular rarefaction and impeded angiogenesis via disturbing mitochondrial function and fatty acid metabolism, which then, leading to adipocytes hypertrophy. Moreover, SIRT3 deficiency predisposed ECs to a SASP phenotype that promoted adipocyte dysfunction and pro-inflammatory macrophages polarization in a paracrine way.

Emerging evidences have proposed a bidirectional model that cardiovascular diseases also contribute to metabolic disorders^4^, ^32^. Here in our study, mice with global or BAT regional endothelium-specific Sirt3 knockout exhibited BAT whitening and accelerated weight gain, as well as worsen glucose intolerance and insulin resistance. Mechanistically, endothelial SIRT3 deficiency resulted in vascular rarefaction and impeded angiogenesis via disturbing mitochondrial function and fatty acid metabolism. Moreover, endothelial SIRT3 deficiency predisposed a SASP phenotype that promoted adipocytes dysfunction and proinflammatory macrophages polarization in a paracrine way. Therefore, the above pathological changes after endothelial SIRT3 loss contributed to diet-induced BAT whitening and obesity progression (Figure 7).

Cardiovascular comorbidities are prevalent in metabolic diseases since local hypoxia, inflammation, and oxidative stress could all deteriorate vascular function and subsequent cardiovascular dysfunction^6^. Reduced angiogenesis not only contributes to an age-related decline in vascular density but also dampens tissue recovery after damage, especially in stroke, ischemia, and peripheral artery disease (PAD)^33^. Additionally, reduced angiogenesis or loss of vascular networks has been suggested to be a primary event preceding metabolic changes in obesity progression^6^. Rebooting angiogenesis has also been validated as an efficient method in improving metabolic dysfunction^4^, ^25^. Nevertheless, excessive or non-targeted angiogenesis might have off-target effects, such as promoting tumor metastasis or exacerbating pulmonary arterial hypertension 34, which therefore suggests the necessity of targeted therapeutic methods. Moreover, ECs in different organs/tissues exhibit heterogeneous phenotypes that fulfill distinct physiological needs^35^. While in the context of obesity, adipose ECs exhibited highest number of differentially expressed genes (DEGs) than liver, kidney, heart, or lung ECs^36^. Here we found global endothelium-specific Sirt3 knockout accelerated the diet-induced obesity process, accompanied by obvious BAT whitening and pathological WAT expansion. Unlike WAT, BAT is highly vascularized and angiogenesis has been indicated to play crucial roles in mediating thermogenesis in case of cold adaptation^37^. Here our results showed that BAT regional endothelium-specific Sirt3 loss not only resulted in vascular rarefaction but also promoted BAT whitening and obesity progression, emphasizing a critical role of BAT endothelial cells in modulating overall metabolism and suggesting that endothelial SIRT3 could be an efficient therapeutic target in modulating angiogenesis and adiposity.

In addition to being an inducer of metabolic diseases, endothelial dysfunction also could be aggravated under metabolic disorders since there is a bidirectional cellular communication that drives endothelial and adipocytes dysfunction reciprocally^6^, ^38^. Insufficient angiogenesis results in localized hypoxia and then, limiting the oxygen supply and subsequent adipocytes death^38^, a pathological process that could further recruit proinflammatory macrophages and facilitate the formation of crown-like structures (CLSs), which ultimately leads to a vicious cycle that leads to adipocyte dysfunction^39^. Additionally, free fatty acids (FFAs) derived from hypertrophic adipocytes exacerbate oxidative stress and endoplasmic reticulum stress that further exacerbates endothelial dysfunction^38^. Therefore, although endothelial Sirt3 was merely knocked out in BAT in our study, the vascular rarefaction was evident in both sWAT and eWAT as well. Therefore, manipulating angiogenesis in BAT could be an efficient approach in preventing the initiation and deterioration of metabolic diseases. In addition to promoting angiogenesis in an organ/tissue way, the optimal intervention timepoint might also make sense. As suggested by single-cell profiling, the proliferation and angiogenesis of adipose ECs were accelerated at the early stage of obesity, while decreased in the context of sustained obesity^36^. Moreover, blocking angiogenesis after the development of obesity also makes sense^40^. Therefore, the on-targeted therapeutic methods of rebooting angiogenesis concerning the optimal occasion and organ/tissue specific levels may need further exploration^38^.

Though representing only 2-5% of the cytosolic volume, mitochondria serve as the signaling and biosynthesis hubs during angiogenesis^13^, ^41^. Mitochondria-derived ROS have been reported to be proangiogenic, while excessive ROS generation due to mitochondrial dysfunction promoted endothelial dysfunction even apoptosis^41^. Here we found that restoring SIRT3 not only alleviated mitochondrial malfunction but also improving the proliferation and migration of ECs, which therefore suggested the significance of mitochondrial homeostasis in mediating angiogenesis. During angiogenesis, metabolic adjustments are desperately needed and ECs shift from a basal metabolic state to a hyperactive status that coordinates both mitochondrial metabolism and fatty acid metabolism for biomass production^13^, ^42^. For instance, the precursors, including oxaloacetate (OAA) and α-ketoglutarate (α-KG), derived from the intact TCA cycle are used in synthesis of lipids, proteins, and nucleotides^13^, ^14^, which therefore indicates that mitochondrial dysfunction or impaired fatty acid metabolism could both impede the angiogenesis. Additionally, adequate endogenous lipid synthesis is indispensable in maintaining the fluidity of membrane and the formation of filopodia and lamellipodia, the essential physiological process in angiogenesis^43^. Here we found that blocking or interfering FASN abolished the proangiogenic effects of SIRT3, emphasizing a critical role of fatty acid synthesis in the formation of capillary-like structures and subsequent angiogenesis, as previously reported^44^. Moreover, blocking the activity or promoting the degradation of FASN could also lead to the accumulation of Mal-CoA, which could also inhibit endothelial proliferation by blocking fatty acid oxidation (FAO) 14, 28, 45. Therefore, blocking both fatty acid synthesis and oxidation abolish the beneficial effects of SIRT3, further demonstrating that the maintenance of normal angiogenesis relies on appropriate mitochondrial function and fatty acid metabolism.

In addition to impeding angiogenesis via disrupting biomass synthesis, mitochondrial dysfunction also contributes to cellular senescence^46^. Similar to the up-regulated inflammatory networks of liver ECs in the context of obesity^36^, here we found PA-treated ECs exhibited a typical SASP phenotype, a pathological process that aggravates inflammation by modulating the recruitment, activation, and function of immune cells^47^. Endothelial senescence not only accounts for age- and disease-associated angiogenesis impairment but also results in pathological changes of adjacent cells in a paracrine way^7^, ^48^. Here we found Sirt3 restoration reduced the release of CASP1, a major component of nucleotide-binding oligomerization domain (NOD)-like receptors that cleaves N-terminal domain of GSDMD, which then, initiating pyroptosis and further evoking inflammation and cell death^49^, ^50^. In addition to pumping out inflammatory cytokines, GSDMD activation also initiates the release of tissue factors and thus, promoting thrombus formation^51^. In contrast to that of macrophages, less attention has been given to the effects of pyroptosis in adipocytes. As suggested by Liu et al., GSDMD activation contributed to LPS-induced adipocytes dysfunction^52^. In addition to punching holes in the plasma membrane, aggravated GSDMD could translocate to mitochondria, permeabilizing the outer membrane and leading to mitochondrial dysfunction^53^, a subcellular event that precedes plasma membrane rupture and predisposes adipocytes to whitening and proinflammatory macrophages polarization^54^. These findings indicate that CASP1 may be a crucial SASP component that contributed to the dysfunction of surrounding cells due to endothelial cell dysfunction.

Rebooting angiogenesis exhibited beneficial effects in preventing obesity and its associated metabolic disorders^4^, ^55^, ^56^. Here in our study, the loss of endothelial Sirt3 exacerbated HFD-induced BAT whitening and obesity progression through impaired angiogenesis and dysregulated paracrine profiles, which, therefore, suggesting the participation of cardiovascular system in modulating overall metabolism. Therefore, the vascular endothelium could be an alternative and accessible therapeutic target in preventing obesity.

While there are still limitations. First, heterogeneity of ECs behaviors across tissues and organs has been validated^57^, therefore, the use of HUVECs in our study might not be sufficient and in situ changes of endothelial behaviors in BAT needed to be further explored. Second, gain of function needed further validation to verify the therapeutic effects of endothelial SIRT3 in treating obesity. Moreover, animal models and cells lines were used in our study, which, however, are quite different in pathological change in human beings. Therefore, further exploration is warranted to confirm the findings in human pathological changes and to promote the translation in clinical practice. Additionally, since different organ-specific ECs exhibit divergent angiocrine profiles and evidences have proven the trans-differentiation of ECs into adipocytes in response to PPARγ activation^16^, ^58^, further exploration considering the organ-specific angiocrine profile and pro-pluripotent effects of ECs are needed.

## Materials and Methods

### Mice

The endothelium-specific Sirt3 knockout mice were obtained using Cre/LoxP system. The F1 generation were constructed using Sirt3^flox/flox^ mice (Jackson Laboratory: 031201) and Tek-Cre mice (Cyagen Biosciences, Inc., China; stock number: C001001). The F1 generation was further crossed with Sirt3^flox/flox^ to generate endothelium-specific Sirt3 knock mice (Sirt3^flox/flox^-Tek-Cre mice, hereafter referred to as Sirt3-EKO mice). The Sirt3-EKO mice and their WT littermates (Sirt3^flox/flox^ mice, hereafter referred to as WT mice) at the age of 8 weeks were fed with HFD (Beijing Huafukang Bioscience, H10045).

To knock out endothelial Sirt3 in BAT, an AAV system based on Cre/LoxP system were performed and the corresponding AAV (AAV-FLT-1 promoter-NLS-Cre-P2A-mScarlet-WPRE, Obiosh, Shanghai) was delivered to BAT directly according to previous protocols^26^. The paraffine sections of BAT were prepared to evaluate the efficiency, as evidenced by the changes of mScarlet signals. Moreover, the knockout efficiency was assessed by the colocalization of mScarlet and SIRT3 signals. After 2 weeks of adaptation, the BAT endothelium-specific Sirt3 knockout mice (Sirt3^flox/flox^-BAT-AAV-Cre mice, hereafter referred to as Sirt3-BAT-EKO mice) and the control mice (hereafter referred to as WT mice) were then fed with HFD.

At the end of intervention, all the mice were anaesthetized using isoflurane (2%) via mask ventilation and then, euthanized via cervical dislocation. All the experimental procedures were performed according to the protocols approved by the committee at Daping Hospital (Army Medical University) and the guidelines of US National Institutes of Health Guide for the Care and Use of Laboratory Animals (8th Edition, 2011).

### Cells

BMDM, HUVECs, 3T3-L1 preadipocytes, primary brown adipocytes, L929 cells, and RAW264.7 cells were used to explore the underlying mechanisms involved. 3T3-L1 preadipocytes were differentiated into terminally differential brown adipocytes according to previous method^59^. Briefly, 3T3-L1 preadipocytes were plated and grown in DMEM supplemented with 10% FBS and 1% penicillin-streptomycin (Pen/Strep) for 2 days. The differentiation was initiated with the incubation with 3-isobutyl-1-methylxanthine (0.5mM, Sigma-Aldrich), triiodothyronine (50nM, Sigma), Rosiglitazone (5μM, Sigma-Aldrich), insulin (5μg/ml, Lily), and Dexamethasone (1μM, Sigma-Aldrich). After 2 days, the incubation medium was removed and replaced with the medium containing triiodothyronine (50nM, Sigma-Aldrich), Rosiglitazone (5μM, Sigma-Aldrich), and insulin (5μg/ml, Lily) for another 6 days.

Bovine serum albumin (BSA, 150μM, Sigma-Aldrich) and palmitic acid (PA, 150μM, Sigma-Aldrich) were used in in vitro experiments. Additionally, 3-TYP (50μM, MCE), C75 (20μM, MCE), etomoxir sodium salt (Eto, 100μM, MCE), malonyl CoA lithium salt (Mal-CoA, 20μM, Sigma-Aldrich), and Z-YVAD-FMK (ZYF, 10μM, Abcam) were incubated simultaneously, respectively. The recombinant lentivirus and plasmids, as well as the corresponding vectors, were generated by Shanghai Genechem Co., Ltd., and were then transfected according to the manufacturers' or Lipofectamine 3000 (Thermo Fisher Scientific), respectively.

The stimulated HUVECs were washed with PBS and then incubated with serum-free DMEM for another 24 hours. Finally, the supernatants were filtered using a filter unit (Millipore) and then applied for the treatment of BMDMs, 3T3-L1 cells, and RAW264.7 cells, respectively.

### BMDMs isolation

L929 cells, an immortalized mouse fibroblast line, were cultured in DMEM supplemented with 10% FBS and 1% penicillin/streptomycin (Gibco, MD, USA) at 37°C in a humidified atmosphere of 5% CO2 and 95% air. After 7 days incubation, the supernatants were collected and filtered for further BMDMs differentiation.

As for BMDMs isolation, the hind legs of above mice were dissected and the bone marrow were harvested and then, cultured with DMEM containing 30% L929 cell supernatant, 20% FBS and 1% Pen/Strep for 5 days to obtain the mature BMDMs^60^.

### ECs isolation

Isolation of ECs in BAT was conducted according to previous study^4^. Briefly, BAT was rapidly removed and digested with the buffer containing collagenase D and dispase II (Roche). The mixture was filtered using a 70μM disposable cell strainer (BD Falcon) and then, centrifuged at 400*g* for 8 minutes at 4 °C. Next, the Dynabeads™ CD31 were added and incubated, following with a sorting using magnetic-activated cell sorting columns.

### Primary brown adipocytes isolation and differentiation

The isolation and differentiation of primary brown adipocytes were conducted according to our previous study^23^. Briefly, interscapular BAT were excised with carefully removing of surrounding muscle and WAT, followed with the digestion in solution that containing Collagenases. Next, the digests were filtered and the stromal vascular fraction cells were isolated using density separation. Finally, the differentiation was initiated with the incubation with 3-isobutyl-1-methylxanthine (0.5mM, Sigma-Aldrich), triiodothyronine (50nM, Sigma-Aldrich), Rosiglitazone (5μM, Sigma-Aldrich), insulin (5μg/ml, Lily), and Dexamethasone (1μM, Sigma-Aldrich). After 2 days, the incubation medium was removed and replaced with the medium containing triiodothyronine (50nM, Sigma‒Aldrich), Rosiglitazone (5μM, Sigma‒Aldrich), and insulin (5μg/ml, Lily) for another 6 days.

### PET/CT scanning

The Micro-PET/CT scanner (Pingseng Healthcare, China) were used to evaluate BAT activities after cold pretreatment. After overnight fasting, all the mice were fasted for 30 minutes at 8 am and then fasted again for 60 minutes. Next, the mice were intraperitoneally injected with ^18^F-FDG (200µCi) and then received cold acclimatization at 4°C for 60 minutes. Finally, the mice were anesthetized using isoflurane (2%) via mask ventilation and then, received PET/CT scanning. The images and data were collected using Avatar 1.5.0 and Recon 1.5.0, respectively. The uptake of ^18^F-FDG was normalized according to body weight.

### Indirect calorimetry

To evaluate the overall metabolism, the Comprehensive Laboratory Animal Monitoring System (CLAMS, Columbus Instruments) were used. The whole-body metabolic states were tested for 2 days after 1 days of adaptation according to the manufacturers' instructions. The consumption of oxygen (Volume O_2_), generation of carbon dioxide (Volume CO_2_), HEAT, as well as respiratory exchange ratio (RER), were determined and normalized by body weight. Additionally, the RER were calculated as VO_2_/VCO_2_.

### Intraperitoneal glucose tolerance test (IPGTT)

The glucose tolerance was assessed using IPGTT. After approximately 16 hours of fasting, all the mice were injected with the glucose solution intraperitoneally according to the body weight (2g glucose/kg). Next, the blood was obtained after cutting the tail and then, placed into the glucose test glucometer at each time point (0, 30, 60, 90, 120, and 180 minutes).

### Insulin tolerance test (ITT)

The insulin sensitivity was evaluated by ITT. Briefly, after approximately 6 hours of fasting, insulin (Novo Nordisk) was injected intraperitoneally according the body weight (0.75IU/kg). Next the blood was obtained after cutting the tail and then, placed into the glucose test glucometer at each time point (0, 30, 60, and 90 minutes).

### Adipose tissue histology

The paraffin sections of BAT and white adipose tissue, including subcutaneous and epididymal adipose tissue (sWAT, eWAT), were prepared and the pathological changes were evaluated through Hematoxylin and eosin (H&E) staining, immunohistochemistry (IHC) staining, and immunofluorescence (IF) staining, respectively.

As for IF staining, the paraffin sections were incubated with primary antibodies, including F4/80 (Abcam, ab6640, 1:400), SIRT3 (CST, C73E3, 1:400), CD31 (Invitrogen, MA3100, 1:200), CD206 (Abcam, ab64693, 1:400), GSDMD (Abcam, ab219800, 1:400), CCR2 (Abcam, ab203128, 1:400), CD11c (Abcam, ab219799, 1:400), and UCP-1 (Invitrogen, SAB1404511, 1:400) at 4°C overnight, respectively. Next, the fluorophore-conjugated secondary antibodies, such as goat anti-rat (Abcam, ab150167, 1:400) and goat anti-rabbit (Invitrogen, A32738, 1:400), were used to incubate the above paraffin sections at 37°C for 60 minutes. In order to evaluate the changes of vascular networks, the GS-IB4 (Invitrogen, I21411, 1:500) were used to incubate the paraffin sections at 37°C for 60 minutes. Finally, the confocal microscopy (A1HD25, Nikon) was used to obtained the images and the Image J were used to obtain quantitative results.

To assess the changes of UCP-1 expression in BAT, the IHC staining was performed. Briefly, the paraffin sections were incubated with UCP-1 (Abcam, ab234430, 1:400) and the following steps were conducted using the SP kit (Beijing ZSJQ Biotechnology Co., Ltd.). Finally, the optical microscope (*Leica*, Germany) was used to obtained the IHC images and the Image J were used to obtain quantitative results.

### Mitochondrial respiration assessment

The Oxygraph-2k were used to assess the changes of mitochondrial respiration according to our previous study^61^. Firstly, the HUVECs were harvested and stabilized in each chamber to record the routine respiration. Next, digitonin (10mg/ml, Sigma-Aldrich) were used to permeabilize the membrane, followed by the successively addition of glutamate (10mM, Sigma-Aldrich), malate (0.5mM, Sigma-Aldrich), and adenosine 5'-diphosphate (5mM, Sigma-Aldrich) to assess the OXPHOS of CI (CI OXPHOS). Next, succinate (10mM, Sigma-Aldrich) and 2-[4- (trifluoromethyl)phenyl hydrazinylidene) propanedinitrile (0.05µM steps, Sigma-Aldrich) were added to evaluate the CI + complex II (CII) OXPHOS and CI + II electron transfer system, respectively. Finally, the rotenone and antimycin A (2.5mM, Sigma-Aldrich) were added successively to assess the electron transfer capacity of CII and residual oxygen consumption, respectively.

### Oil Red O staining

The accumulation of lipid droplets was assessed using the Oil red O staining. Briefly, the terminally differentiated 3T3-L1 adipocyte and frozen sections of tissues were fixed and then, washed with PBS for three times. Next, Oil red O solution was added and incubated for 10 minutes, followed with the decontamination of 60% isopropanol. Finally, the optical microscope was used to obtained the images (*Leica*, Germany).

### EdU staining

The changes of proliferation process were assessed using the Click-iT™ EdU Imagine Kit (Invitrogen, C10340). Firstly, 3.7% formaldehyde were used to fix the stimulated ECs. Next, the reaction buffer was prepared and then, applied to plate. After incubation of 30 minutes, Hoechst 33342 was added. Finally, the images and the quantitative results were obtained using confocal microscope (A A1HD25, *Nikon*) and Image J, respectively.

### F-actin staining

The formation of F-actin was measured using Phalloidin-iFluor 647 (Abcam, ab176795). Firstly, 4% formaldehyde were used to fix the stimulated HUVECs, followed by the permeabilization using 0.1% Triton X-100. After incubation with working solution for 90 minutes, the confocal microscope was used to obtained the images (A1HD25, *Nikon*).

### Transwell migration

The transwell chamber were used to evaluate the changes of migration (Corning, #3428). Firstly, 1 × 10^5^ of ECs that suspended in serum-free DMEM were added in the upper chambers, while DMEM with 10% FBS were added in lower chambers. After incubation of 24 hours, ECs in the lower chambers were fixed and stained with crystal violet (Beyotime, #C0121). Finally, the optical microscope was used to quantify the migrated cells (*Leica*, Germany).

### ROS measurements

The DHE (Invitrogen, D11347) and MitoSox (Invitrogen, M36008) were used to measure the production of cytosolic and mitochondrial ROS, respectively. Briefly, the pretreated HUVECs were incubated with serum-free DEME containing DHE (5μM) or MitoSox (5μM) at 37°C for 30 minutes in dark, respectively. Finally, the images and quantitative results were obtained using fluorescence microscope (TE2000, *Nikon*) and FluoroSkan Ascent Fluorometer (Thermo Fisher Scientific), respectively.

### Mitochondrial calcium assessment

The Rhod-2 AM (Invitrogen, R1244) were used to measure the changes of mitochondrial Ca^2+^ uptake according to our previous study^61^. Briefly, the pretreated ECs were incubated with Ca^2+^-free solution containing Rood-2 AM (5μM) at 37°C for 30 minutes in dark and then, permeabilized with digitonin. Next, the incubated HUVECs were washed with to remove the extraneous dye. Mitochondrial Ca^2+^ uptake were detected after stimulation by ATP (300μM, Sigma-Aldrich) using the microplate reader (Thermo Fisher Scientific).

### Senescence β-Galactosidase staining

The β-Galactosidase staining kit were used to evaluate the changes of senescence (CST, 9860S). Briefly, the pretreated ECs were fixed with Fixative Solution for 15 minutes at room temperature and then incubated using the β-Galactosidase Staining Solution at 37°C overnight in a dry incubator (No CO_2_). Finally, the optical microscope was used to obtained the images (*Leica*, Germany).

### Tube formation assay

The angiogenesis was evaluated by tube formation assay according to previous study^62^. Briefly, the pretreated ECs were seeded in 24-well culture plates (1×10^4^/well) that precoated with Matrigel (Corning, #356234). After 24 hours, the images were acquired with an inverted microscope and quantitative results were obtained using Image J.

### Cell Counting Kit-8 assay

The cell viability was assessed using Cell Counting Kit-8 assay (Beyotime, C0041). Briefly, BMDMs, RAW264.7 cells, and brown 3T3-L1 adipocytes were plated in 96-well plates (0.5×10^4^/well) and incubated with 10μl CCK8 at 37°C for 3 hours, respectively. Finally, OD at 450nm were determined using the microplate reader (Thermo Fisher Scientific).

### Quantitative Real-time PCR

Total RNA was extracted using TRIzol (Invitrogen) according to the manufacturer's instructions, as indicated in our previous study^61^. The isolated RNA was reverse-transcribed into cDNA via a Transcriptor First-Strand cDNA Synthesis Kit (Roche). A SYBR Green RT-PCR Kit (Roche) was used for the amplification reactions according to the three-step protocol described by the manufacturer (Bio-Rad, USA). The data were normalized to that of β-actin. The primers used are listed in Supplementary Table 1.

### Immunoprecipitation (IP)

The acetylation levels of FASN were measured using IP according to our previous study^61^. Briefly, ECs were lysed with RIPA buffer (1% Triton-X, 0.1% SDS, 0.5% deoxycholate, 50mM Tris pH 7.5, 150mM NaCl, protease inhibitor, Roche) and then, incubated with an anti-FASN antibody (Abcam, ab128870) or equal amount of IgG (CST, 2729S). Next, the Dynabeads Protein G (Invitrogen, 10004D) were added and the mixture were incubated at 4°C overnight and then, washed with the lysis buffer for at least three times. Finally, the proteins were eluted with loading buffer at 100°C for 8 min and further analyzed via Western blotting.

### Western blot

The cells or tissues were lysed using the RIPA lysis buffer according to our previous study^61^. Briefly, the protein concentrations were determined by bicinchoninic acid assay (BCA) method and equal amount of protein were separated by SDS-PAGE and transferred onto polyvinylidene difluoride membranes (Millipore). After blocking, the filters were incubated with following primary antibodies, including SIRT3 (CST, 2627S), IL-6 (CST, 12912S), IL-1β (CST, 12703S), FASN (Abcam, ab128870), ACCS (CST, 3676S), VCAM (Abcam, ab134047), ACLY (Invitrogen, MA5-24861), and CPT-1a (Abcam, ab220789) at 4°C overnight, followed with the incubation with the appropriate horseradish peroxidase-conjugated secondary antibody (Santa Cruz Biotechnology). Finally, the immune complexes were visualized using a chemiluminescence horseradish peroxidase substrate (WBKLS0100, Millipore).

### HPIC-MS/MS

Targeted metabolism was performed by Shanghai BIOTREE Biological Technology Co., Ltd. (China). Briefly, a total of 1×10^6^ ECs were collected and the metabolites were extracted after repeated frozen and thaw in liquid nitrogen, vortexed and sonicated in MeOH, incubation at -40°C and centrifugation. Next, the protein concentrations were measured and the supernatants were dried and reconstituted, followed by filtration and transferation into injection vials for further HPIC-MS/MS analysis (Thermo Scientific Dionex ICS-6000 HPIC system).

### RNA sequence

The RAN were isolated using TRIzol reagent (Invitrogen) and the sequencing were performed by y KnoriGene Co., Ltd. (China). Briefly, after assessment of purity, quantity, and integrity, the RNA was fragmented and then, reverse-transcribed into cDNA. RNA Library were constructed using NEBNext® Ultra™ RNA Library Prep Kit for Illumina® and the sequencing were performed using Illumina NovaSeq PE150.

### Proteomics

The stimulated ECs were washed with PBS and then cultured with serum-free DMEM for another 24 hours. Supernatants were collected and clarified at 1,000rpm and 12,000rpm for 10 minutes. Next, proteomic-based global secretome analysis was performed by PTM BioLab, Inc. (Hangzhou, China). Briefly, the cellular debris was further removed by centrifugation at 12,000g at 4°C and the top 14 high abundance proteins were removed, followed by protein concentration determinant by BCA kit. Next, the protein solution was digested and derived peptides were desalted by C18 SPE column, followed by the labelling using TMT reagent (Thermo Fisher Scientific) and subsequent desalting with Strata X C18 SPE column (Phenomenex). Finally, LC-MS/MS analysis was performed and MaxQuant search engine was used to analyzed the data (v.1.6.15.0).

### Statistics

Quantitative results are expressed as the means ± SEM. The differences between two groups were analyzed using two-tailed Student's *t* tests. The differences among three or more groups were analyzed using one-way ANOVA followed by Tukey *post hoc* tests for multiple comparisons. Two-way ANOVA was used for repeated measurements of BW. The area under the curve (AUC) was calculated for the IPGTT and ITT for multiple measurements. Graphs were created using Prism 8.0 (GraphPad Software), and statistical analysis was performed with GraphPad Prism. A P value < 0.05 was considered to indicate statistical significance.

## Supplementary Material

Supplementary figures and tables.
