# Supplementary material for: Endothelial SIRT3 deficiency predisposes brown adipose tissue to whitening in diet-induced obesity
Source: Int J Biol Sci. 2025 May 15;21(8):3444–60. doi: 10.7150/ijbs.110741 (PMC12160546; doi:10.7150/ijbs.110741)
Supplement: Supplementary file 1 — Supplementary figures and tables. [file ijbsv21p3444s1.pdf]

Figure S1. Endothelial SIRT3 deficiency decreased overall metabolism.

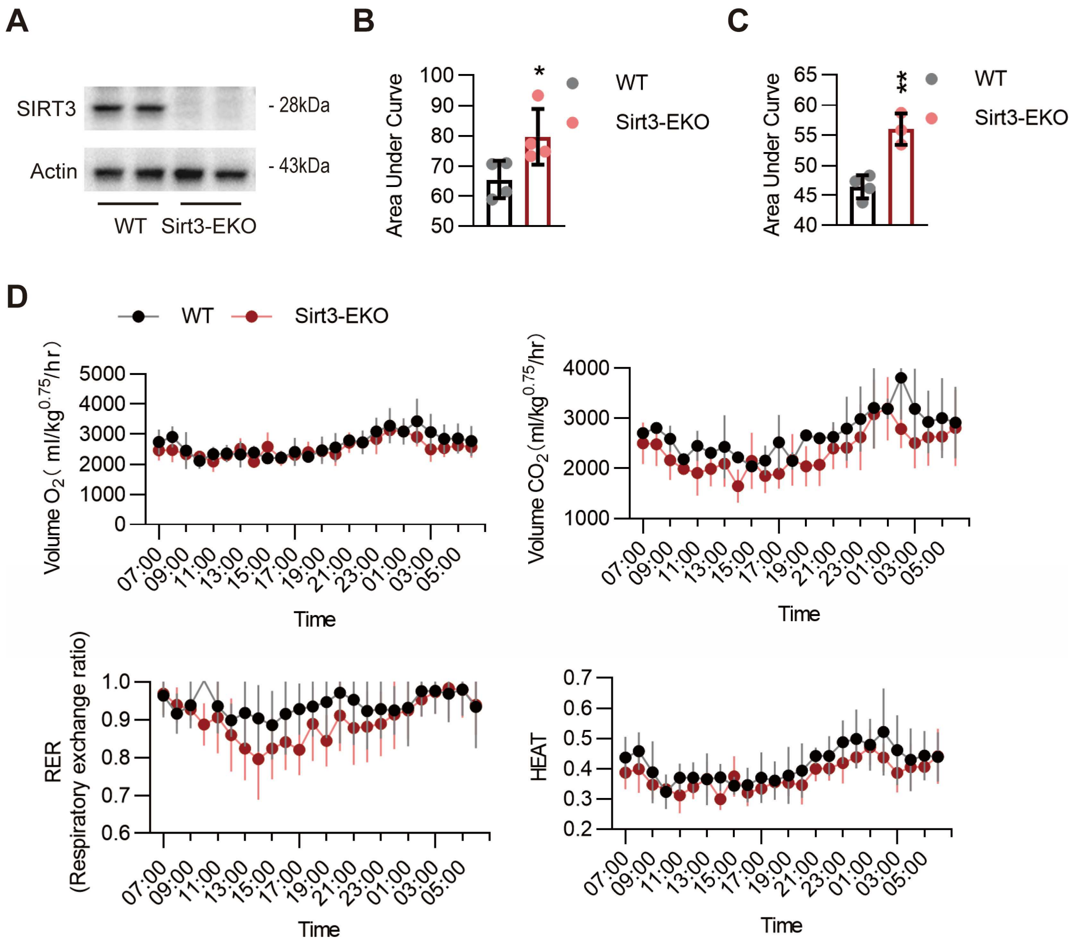

(A) Representative images of Western blots of SIRT3 in isolated ECs of Sirt3$^{flox/flox}$ mice (hereafter referred to as WT mice) and Sirt3$^{flox/flox}$-Tek-Cre mice (hereafter referred to as Sirt3-EKO mice). β-actin served as a loading control. **(B-C)** The changes of Area under Curve (AUC) of IPGTT (**B**, n=4) and ITT (**C**, n=3 or n=4) in WT and Sirt3-EKO mice fed with HFD (n=4 or 3). **(D)** Volume O_2, Volume CO_2, RER, and Heat of WT and Sirt3-EKO mice fed with HFD, detected by CLAMS (n=4). ECs: endothelial cells; HFD: high fat diet; Volume O2: consumption of Oxygen; Volume CO2: generation of carbon dioxide; RER: respiratory exchange ratio. IPGTT: Intraperitoneal glucose tolerance test; ITT: insulin tolerance test.

Figure S2. The BAT regional endothelium-specific Sirt3 knockout exacerbated HFD-induced metabolic disorders.

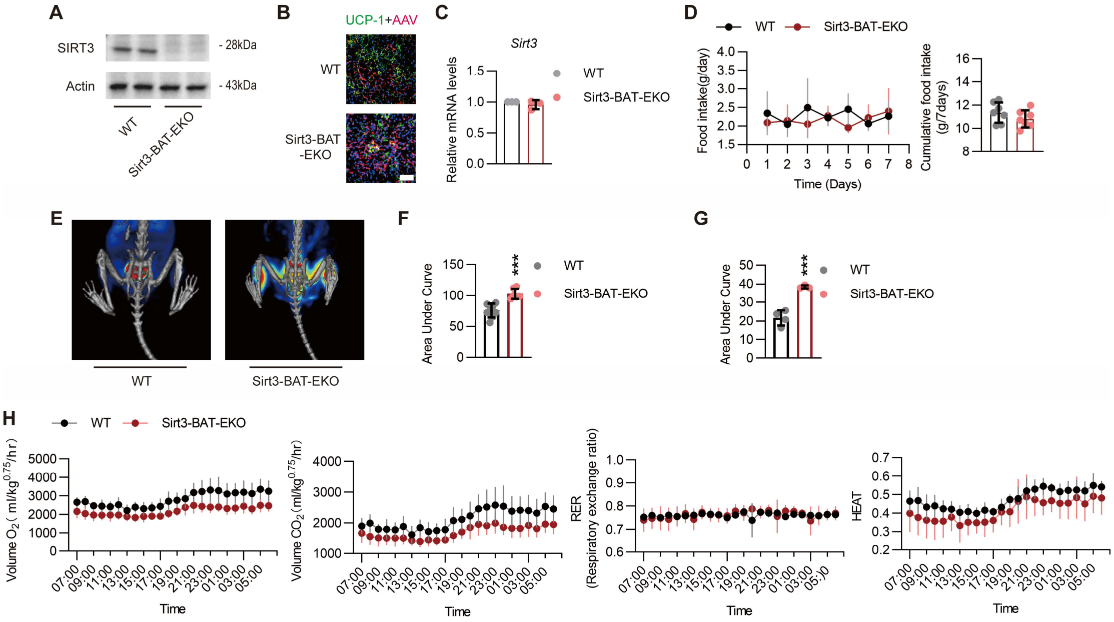

(**A**) Representative images of Western blots of SIRT3 in isolated ECs of BAT from regional-specific endothelium Sirt3 knockout mice (hereafter referred to as Sirt3-BAT-EKO mice) and their control mice (hereafter referred to as WT mice). β-actin served as a loading control. **(B)** Representative images of IF staining of AAV (Red) and UCP-1 (Green) in BAT from WT and Sirt3-BAT-EKO mice (n=4). Scales bars, 50μm. (**C**) The mRNA levels of Sirt3 in isolated brown adipocytes of WT and Sirt3-BAT-EKO mice (n=3). β-actin served as a loading control. (D) The cumulative food intake of WT and Sirt3-BAT-EKO mice fed with HFD that were monitored for 7 days at the end of HFD treatment (n=5). (**E**) Representative images of ^{18}F-FDG uptake in the skeletal muscle of WT and Sirt3-BAT-EKO mice, detected by PET-CT (n=3 or n=4). **(F-G)** The changes of Area under Curve (AUC) of IPGTT (**F**, n=6 or n=8) and ITT (**G**, n=3 or n=4) in WT and Sirt3-EKO mice fed with HFD. (**H**) Volume O_2, Volume CO_2, RER, and Heat of WT and Sirt3-EKO mice fed with HFD, detected by CLAMS (n=4). (**D**) Representative images of ^{18}F-FDG uptake in the skeletal muscle of WT and Sirt3-BAT-EKO

mice, detected by PET-CT (n=3 or n=4). IF: immunofluorescence; AAV: Adeno-associated virus; HFD: high fat diet; Volume O2: consumption of Oxygen; Volume CO2: generation of carbon dioxide; RER: respiratory exchange ratio; IPGTT: Intraperitoneal glucose tolerance test; ITT: insulin tolerance test.

Figure S3. Endothelial SIRT3 deficiency exacerbated PA-induced senescence.

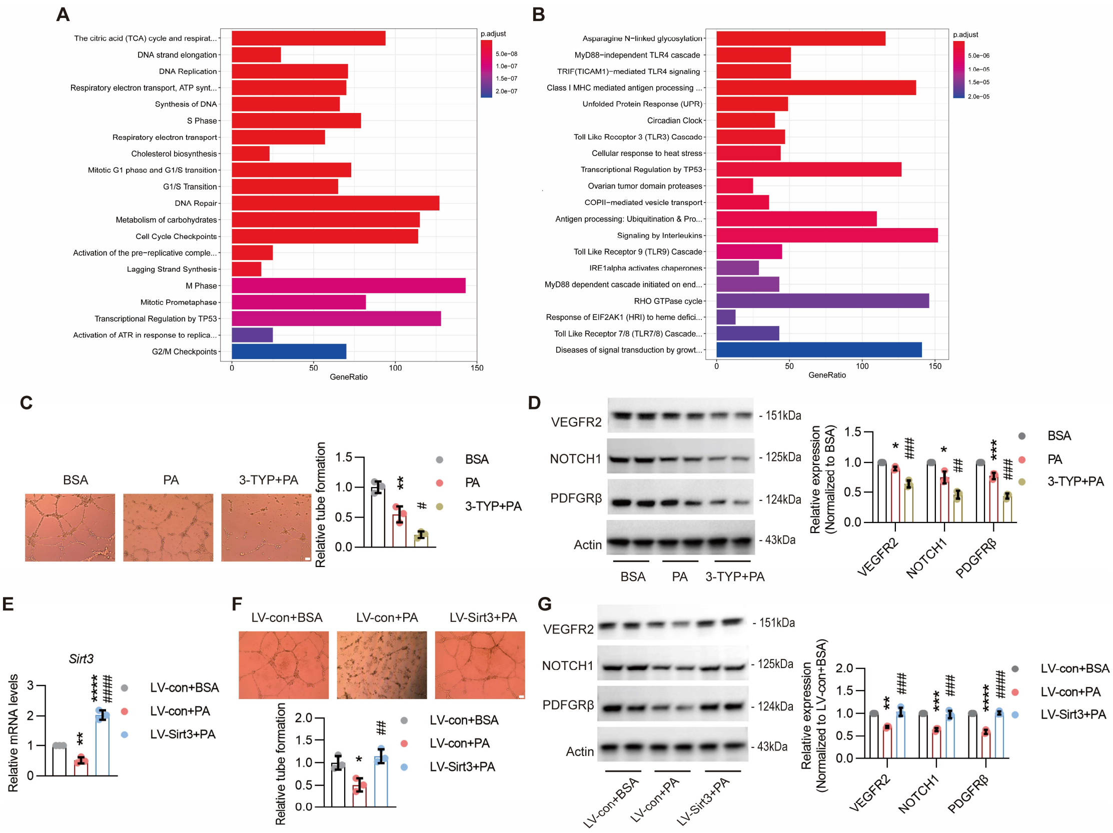

(**A–B**) Representative images of down-regulated (**A**) and up-regulated (**B**) pathways in ECs treated with BSA or PA, detected by RNA sequencing (n=3). (**C-D**) Representative images and quantitative results of tube formation (**C**), as wells as Western blots and quantitative results of VEGFR2, NOTCH1, and PDGFRβ (**D**), in ECs that treated with BSA, PA, or 3-TYP+PA (n=3). β-actin served as a loading control. Scales bars, 50μm. (**E-G**) The mRNA levels of Sirt3 (**E**), tube formation and quantitative results. (**F**), and Western blots and quantitative results of

VEGFR2, NOTCH1, and PDGFRβ (**G**), in ECs that treated with LV-con+BSA, LV-con+PA, or LV-Sirt3+PA (n=3). β-actin served as a loading control. Scales bars, 50μm. $^{*}P< 0.05$, $^{**}P< 0.01$, $^{***}P< 0.001$, $^{****}P< 0.0001$ compared with BSA or LV-con + BSA (**C**, **D**, **E, F, G**). $^{\#}P< 0.05$, $^{\#\#}P< 0.01$, $^{\#\#\#}P< 0.001$, $^{\#\#\#\#}P< 0.0001$ compared with PA or LV-con + PA (**C**, **D**, **E, F, G**). BSA: bovine serum albumin; PA: palmitic acid; VEGFR2: Vascular Endothelial Growth Factor Receptor 2; NOTCH1: Notch Receptor 1; PDGFRβ: platelet-derived growth factor receptor β.

Figure S4. The modulatory effects of endothelial SIRT3 in the mitochondrial metabolite profile.

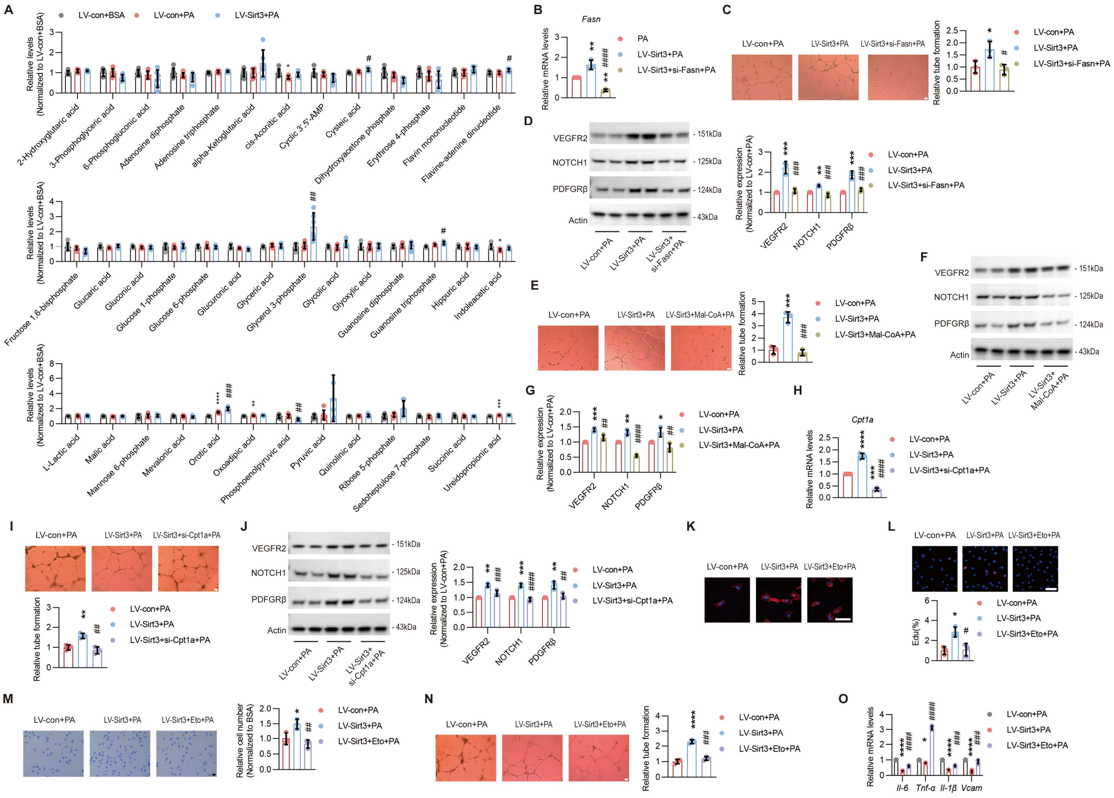

(**A**) The changes of mitochondrial metabolites profile of ECs treated with LV-con+BSA, LV-con+PA, or LV-Sirt3+PA, detected by HPIC-MS/MS (n=6). The data were normalized to LV-con+BSA. (**B-D**) The mRNA levels of *Fasn* (**B**), tube formation and quantitative results (**C**), and Western blots and quantitative results of of VEGFR2, NOTCH1, and PDGFRβ (**D**), in ECs

that treated with LV-con+PA, LV-Sirt3+PA, or LV-Sirt3+si-Fasn+PA (n=3). β-actin served as a loading control. Scales bars, 50μm. (**E-G**) Representative images and quantitative results of tube formation (**E**), as well as Western blots and quantitative results of VEGFR2, NOTCH1, and PDGFRβ (**F-G**), in ECs treated with LV-con+PA, LV-Sirt3+PA, or LV-Sirt3+Mal-CoA+PA (n=3). β-actin served as a loading control. Scales bars, 50μm. (**H-J**) The mRNA levels of Cpt-1a (**H**), tube formation and quantitative results (**I**), and Western blots and quantitative results of of VEGFR2, NOTCH1, and PDGFRβ (**J**), in ECs that treated with LV-con+PA, LV-Sirt3+PA, or LV-Sirt3+si-Cpt1a+PA (n=3). β-actin served as a loading control. Scales bars, 50μm. (**E-F**) The mRNA levels of *Cpt1a* (**E,** n=3), tube formation and quantitative results (**F,** n=3) of ECs that treated with LV-con+PA, LV-Sirt+PA, or LV-Sirt3+si-Cpt1a+PA (n=3). Scales bars, 50μm. (**K-O**) Representative images and quantitative results of F-actin staining (**K**), EdU staining (**L**), transwell staining (**M**), tube formation and quantitative results (**N**), and mRNA levels of *Il-6*, *Tnf-a, Il-1β*, and *Vcam* (**O**) in ECs treated with LV-con+PA, LV-Sirt3+PA, or LV-Sirt3+ Eto (100μM) + PA (n=3). β-actin served as a loading control. Scales bars, 50μm. $^{*}P< 0.05$, $^{**}P< 0.01$, $^{***}P< 0.001$, $^{****}P< 0.0001$ compared with LV-con + PA (**B, C, D, E, F, G, H, I, J, L, M, N, O**). $^{\#}P< 0.05$, $^{\#\#}P< 0.01$, $^{\#\#\#}P< 0.001$, $^{\#\#\#\#}P< 0.0001$ compared with LV-Sirt3 + PA (**B, C, D, E, F, G, H, I, J, L, M, N, O**). FASN: Fatty Acid Synthase; BSA: bovine serum albumin; PA: palmitic acid; Mal-CoA: Malonyl-CoA; CPT-1a: Carnitine Palmitoyltransferase 1A; Eto: Etomoxir sodium salt; VEGFR2: Vascular Endothelial Growth Factor Receptor 2; NOTCH1: Notch Receptor 1; PDGFRβ: platelet-derived growth factor receptor β; IL-6: Interleukin 6; TNF-α: Tumor Necrosis Factor; IL-1β: Interleukin 1β; VCAM: Vascular cell adhesion molecule.

Figure S5. The paracrine effects of ECs in regulating adipocyte function and pro-inflammatory macrophages polarization.

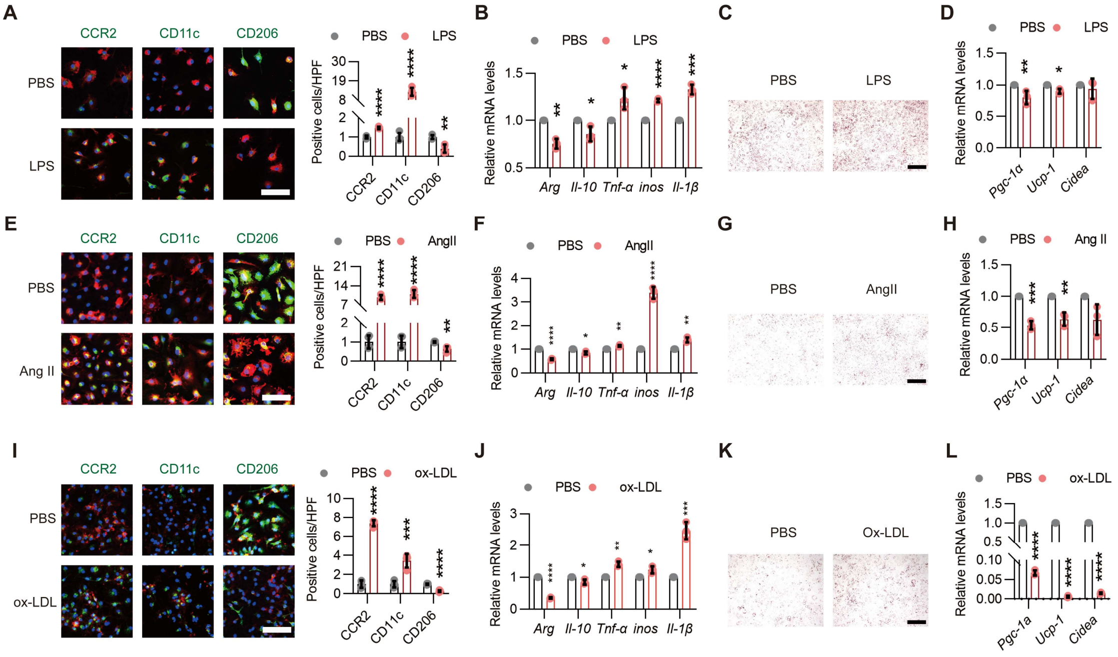

(**A**) Representative images of IF staining (left) and quantitative results (right) of F4/80 (Red), CD11c (Green), CCR2 (Green), and CD206 (Green) in BMDMs treated with the CM of PBS- or LPS-pretreated ECs (n=3). Scales bars, 50μm. (**B**) The mRNA levels of *Arg*, *Il-10*, *Tnf-α*, *inos*, and *Il-1β* in BMDMs treated with the CM of PBS- or LPS-pretreated HUVECs (n=3). β-actin served as a loading control. (**C-D**) Representative images of lipid droplets formation **(C)** and the mRNA levels of *Ucp-1*, *Pgc-1α*, and *Cidea* **(D)** in terminally differential brown 3T3-L1 adipocytes treated with the CM of PBS- or LPS-pretreated ECs (n=3). Scales bars, 200μm. β-actin served as a loading control. (**E**) Representative images of IF staining (left) and quantitative results (right) of F4/80 (Red), CD11c (Green), CCR2 (Green), and CD206 (Green) in BMDMs treated with the CM of PBS or AngII-pretreated ECs (n=3). Scales bars, 50μm. (**F**) The mRNA levels of *Arg*, *Il-10*, *Tnf-α*, *inos*, and *Il-1β* in BMDMs treated with the CM of PBS- or AngII-pretreated ECs (n=3). β-actin served as a loading control. (**G-H**) Representative

images of lipid droplets formation **(G)** and the mRNA levels of *Ucp-1*, *Pgc-1α*, and *Cidea* **(H)** in terminal differential brown 3T3-L1 adipocytes treated with the CM of PBS- or AngII-pretreated ECs (n=3). Scales bars, 200μm. β-actin served as a loading control. (**I**) Representative images of IF staining (left) and quantitative results (right) of F4/80 (Red), CD11c (Green), CCR2 (Green), and CD206 (Green) in BMDMs treated with CM of PBS- or ox-LDL-pretreated ECs (n=3). Scales bars, 50μm. (**J**) The mRNA levels of *Arg*, *Il-10*, *Tnf-α*, *inos*, and *Il-1β* in BMDMs treated with the CM of PBS- or ox-LDL-pretreated ECs (n=3). β-actin served as a loading control. (**K-L**) Representative images of lipid droplets formation **(K)** and the mRNA levels of *Ucp-1*, *Pgc-1α*, and *Cidea* **(L)** in terminal differential brown 3T3-L1 adipocytes treated with the CM of PBS- or ox-LDL-pretreated HUVECs (n=3). Scales bars, 200μm. β-actin served as a loading control. $^{*}P< 0.05$, $^{**}P< 0.01$, $^{***}P< 0.001$, $^{****}P< 0.0001$ compared with PBS (**A**, **B**, **D**, **E**, **F**, **H**, **I**, **J**, **L**). BMDMs: bone marrow derived macrophages; CM: conditional medium; LPS: Lipopolysaccharide Arginase; IL-10: Interleukin 10; iNOS: inducible Nitric oxide synthase; TNF-α: Tumor Necrosis Factor; IL-1β: Interleukin 1β; PBS: phosphate buffer saline; UCP-1: uncoupling protein 1; PGC-1α: Peroxisome proliferator-activated receptor-gamma coactivator 1 α; Cidea: Cell death inducing DFFA like effector A; AngII: Angiotensin II; ox-LDL: Oxidized low-density lipoprotein.

Figure S6. The effects of endothelial SIRT3 in regulating the production of angiocrine factors.

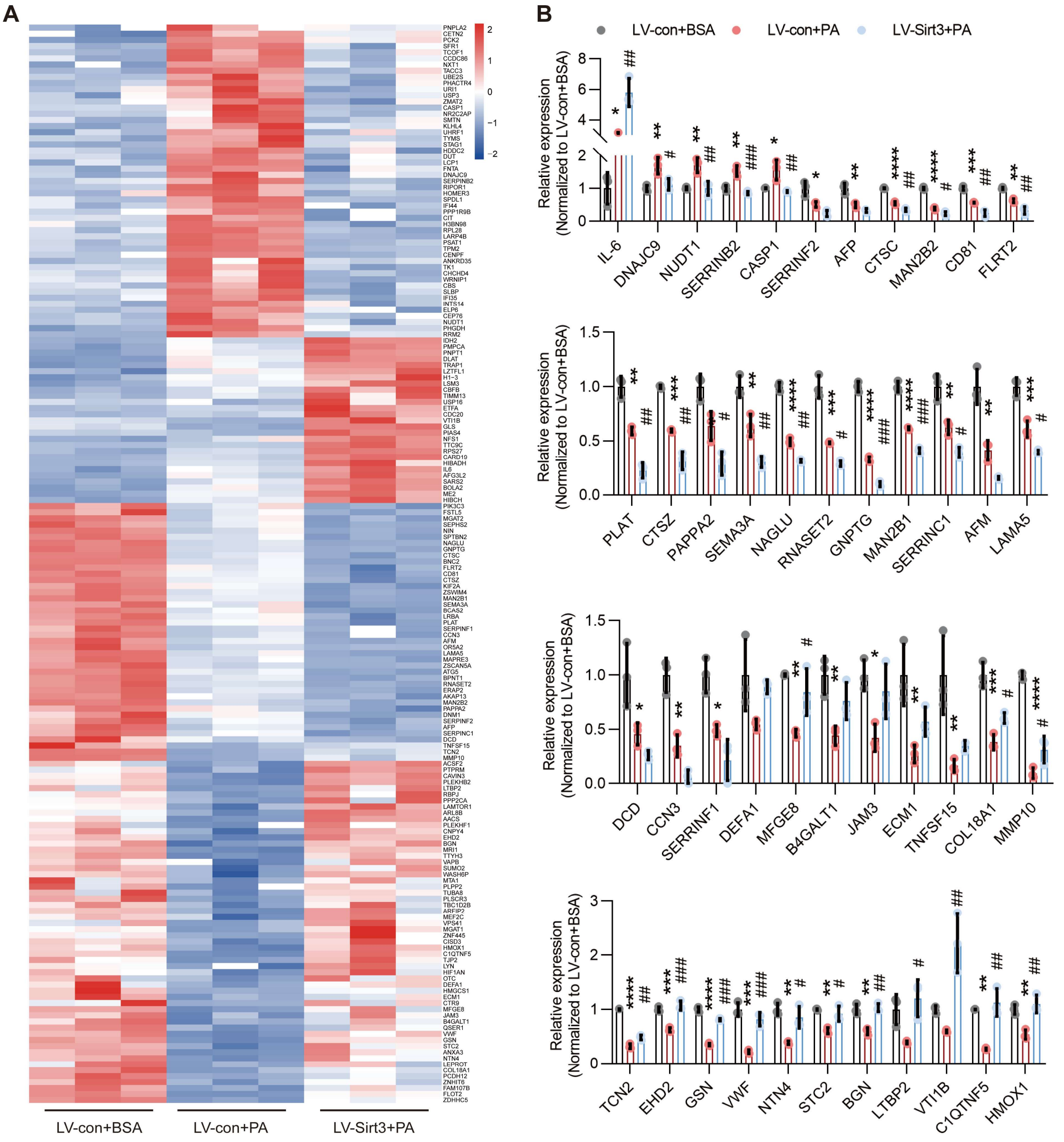

(A) The Heap map of angiocrine proteomic data ECs treated with LV-con+BSA, LV-con+PA, or LV-Sirt3+PA (n=3). **(B)** The quantification of secretory proteins in ECs treated with LV-con+BSA, LV-con+PA, or LV-Sirt3+PA, detected by proteomics (n=3). $^{*}P$< 0.05, $^{**}P$< 0.01, $^{***}P$< 0.001, $^{****}P$< 0.0001 compared with LV-con+BSA (**B**). $^{\#}P$< 0.05, $^{\#\#}P$< 0.01, $^{\#\#\#}P$< 0.001, $^{\#\#\#\#}P$< 0.0001 compared with LV-con+PA (**B**). BSA: bovine serum albumin; PA: palmitic acid.

Figure S7. The angiocrine effects of ECs in modulating viability of adipocytes and macrophages.

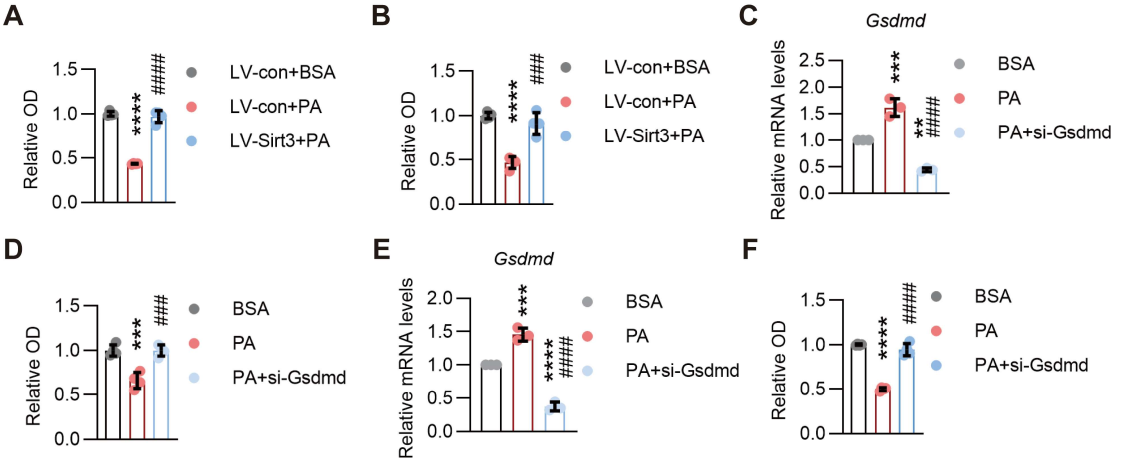

(A-B) The cell viability of terminally differential brown 3T3-L1 adipocytes (**A**) or BMDMs (**B**) that treated with the CM of LV-con+BSA-, LV-con+PA-, or LV-Sirt3+PA-pretreated ECs, assessed by CCK8 measurement (n=3). **(C-D)** The mRNA levels of Gsdmd and cell viability of si-con or si-Gsdmd-pretreated terminally differential brown 3T3-L1 adipocytes that treated with the CM of BSA- or PA-pretreated ECs (n=3). **(E-F)** The mRNA levels of Gsdmd and cell viability of si-con or si-Gsdmd-pretreated RAW264.7 cells that treated with the CM of BSA- or PA-pretreated ECs (n=3). $^{***}P< 0.001$, $^{****}P< 0.0001$ compared with LV-con+BSA (**A-B**) or BSA (**C-D**). $^{\#}P< 0.05$, $^{\#\#}P< 0.01$, $^{\#\#\#}P< 0.001$, $^{\#\#\#\#}P< 0.0001$ compared with LV-con+PA (**A-B**) or PA (**C-D**). BSA: bovine serum albumin; PA: palmitic acid; GSDMD: Gasdermin D.

Table S1. Primers used for qRT–PCR in this study.

Human *β-actin*-forward	ACACCCTTTCTTGACAAAACCT
Human *β-actin* -reverse	CGCATCTCATATTTGGAATGACT
Human *Il-1β*-forward	TTCGACACATGGGATAACGAGG
Human *Il-1β*-reverse	TTTTTGCTGTGAGTCCCGGAG
Human *Vcam*-forward	CGAAAGGCCCAGTTGAAGGA
Human *Vcam*-reverse	GAGCACGAGAAGCTCAGGAGAAA
Human *Il-6*-forward	CCTGAACCTTCCAAAGATGGC
Human *Il-6*-reverse	TTCACCAGGCAAGTCTCCTCA
Human *Tnf-α*-forward	GAGGCCAAGCCCTGGTATG
Human *Tnf-α*-reverse	CGGGCCGATTGATCTCAGC
Human *Fasn*-forward	GTGTGGACATGGTCACAGATG
Human *Fasn*--reverse	GACCGCTTGGGTAATCCATA
Human *Acc*-forward	GCTTCTTTCCCATTCTTCGG
Human *Acc*-reverse	CCCGGACTCATTCAGGATTG
Human *Acly* forward	TTCGTCAAACAGCACTTCC
Human *Acly* -reverse	ATTTGGCTTCTTGGAGGTG
mouse *β-actin*-forward	TCCATCATGAAGTGTGACGT
mouse *β-actin*-reverse	TACTCCTGCTTGCTGATCCAC
mouse *Arg*-forward	GGGCAACCTGTGTCCTTTCTCC
mouse *Arg*-reverse	GGTCTACGTCTCGCAAGCCAAT

mouse *Il-10*-forward	GGGTTGCCAAGCCTTATCGGAA
mouse *Il-10*-reverse	CTTCACCTGCTCCACTGCCTTG
mouse *Tnf-α*-forward	GGAACTGGCAGAAGAGGCACTC
mouse *Tnf-α*-reverse	GTAGACAGAAGAGCGTGGTGGC
mouse *inos*-forward	CCCTCCTCGTTCAGCTCACCTT
mouse *inos*-reverse	CCGCTCTCATCCAGAACCTCCA
mouse *Il-1β*-forward	CCTGTGTCTTTCCCGTGGACCT
mouse *Il-1β*-reverse	TCGGAGCCTGTAGTGCAGTTGT
mouse *Ucp-1*-forward	GCGGGCATTCAGAGGCAAATCA
mouse *Ucp-1*-reverse	TGTTTCCGAGAGAGGCAGGTGT
mouse *Pgc-1α*-forward	GTAAATCTGCGGGATGATGG
mouse *Pgc-1α*-reverse	AGCAGGGTCAAAATCGTCTG
mouse *Cidea*-forward	ATCACAACTGGCCTGGTTACG
mouse *Cidea*-reverse	TACTACCCGGTGTCCATTTCT
mouse *Il-18*-forward	ATGCTTTCTGGACTCCTGCC
mouse *Il-18*-reverse	AGTCTTCTGACATGGCAGCC
mouse *Gsdmd*-forward	TCGGCAGGGGTGAAAAATC
mouse *Gsdmd*-reverse	AATGTTCCCATCGACGACAT
